# Optical tweezer and TIRF microscopy for single molecule manipulation of RNA/DNA nanostructures including their rubbery property and single molecule counting

**DOI:** 10.52601/bpr.2021.210003

**Published:** 2021-12-31

**Authors:** Chiran Ghimire, Peixuan Guo

**Affiliations:** 1 Center for RNA Nanobiotechnology and Nanomedicine; College of Pharmacy; Dorothy M. Davis Heart and Lung Research Institute; College of Medicine; James Compréhensive Cancer Center, The Ohio State University, Columbus, OH 43210, USA

**Keywords:** Stoichiometry, pRNA, Photobleaching, Bacteriophage Phi29, DNA packaging motor, Portal vertex, Passive targeting, Cancer imaging, Renal excretion, Organ accumulation, Single layer patterned arrays

## Abstract

Life science is often focused on the microscopic level. Single-molecule technology has been used to observe components at the micro- or nanoscale. Single-molecule imaging provides unprecedented information about the behavior of individual molecules in contrast to the information from ensemble methods that average the information of many molecules in various states. A typical feature of living systems is motion. The lack of synchronicity of motion biomachines in living systems makes it challenging to image the motion process with high resolution. Thus, single-molecule technology is especially useful for real-time study on motion mechanism of biomachines, such as viral DNA packaging motor, or other ATPases. The most common optical instrumentations in single-molecule studies are optical tweezers and single molecule total internal refection fluorescence microscopy (smTIRF). Optical tweezers are the force-based technique. The analysis of RNA using optical tweezer has led to the discovery of the rubbery or amoeba property of RNA nanoparticles for compelling vessel extravasation to enhance tumor targeting and fast renal excretion. The rubbery property of RNA lends mechanistic evidence for RNAs use as an ideal reagent in cancer treatment with undetectable toxicity. Single molecule photobleaching allows for the direct counting of biomolecules. This technique was invented for single molecule counting of RNA in the phi29 DNA packaging motor to resolve the debate between five and six copies of RNA in the motor. The technology has subsequently extended to counting components in biological machines composed of protein, DNA, and other macromolecules. In combination with statistical analysis, it reveals biomolecular mechanisms in detail and leads to the development of ultra-sensitive sensors in diagnosis and forensics. This review focuses on the applications of optical tweezers and fluorescence-based techniques as single-molecule technologies to resolve mechanistic questions related to RNA and DNA nanostructures.

## INTRODUCTION

Optical tweezers (Ashkin 1986; Moffitt *et al*. 2008), magnetic tweezers (Neuman and Nagy 2008), super-resolution microscopy (Schermelleh *et al*. 2019), single-molecule total internal reflection fluorescence (TIRF) microscopy (Kudalkar *et al*. 2016; Rueda and Walter 2005), and other techniques based on force, fluorescence (Friedman and Gelles 2015), electricity (Cockroft *et al*. 2008), or their combinations (Whitley *et al*. 2017) have been recognized as unique methods for research related to singular cells, living specimens, cell organelles, and even single-molecules such as RNA, DNA, protein, and other macromolecules (Adachi *et al*. 2007; Borodavka *et al*. 2012; Funatsu *et al*. 1995; Koyama-Honda *et al*. 2005; Mehta *et al*. 2016; Milstein *et al*. 2012; Newby Lambert *et al*. 2006). The single-molecule technology provides an alternative set of approaches to conventional techniques for the delivery of vital information about biological processes at the level of molecular movements, dynamics, and functions, which play significant roles in many scientific fields such as drug discovery research and development (Hashemi Shabestari *et al*. 2017; Myong *et al*. 2006). Many optical-related techniques can be applied to biological research at the single-molecule level, including but not limited to optical tweezers and fluorescence-related techniques such as FRET and single-molecule photobleaching assay (Fairman-Williams and Jankowsky 2012; Hashemi Shabestari *et al*. 2017). Single-molecule techniques have a resolution up to sub-nanometer in distance measurements, sub-piconewton in force measurements, and microsecond in time measurements (Neuman *et al*. 2008). A typical feature of living systems is motion. The lack of synchronicity of motion machines in living systems makes it challenging to analyze the motion process with high resolution. Thus, single-molecule technology is especially important in the analysis of motion machines. Developments over the past few decades demonstrate that single-molecule manipulation technology has already facilitated many groundbreaking achievements pertaining to basic life science questions. Further, the potential applications of these methods in biology research show additional broad possibilities. The single-molecule technology can obtain abundant information on an individual molecule other than the averaged information from many molecules in various states acquired from ensemble methods, which assists in elucidating basic life science-related questions to accelerate many pioneering accomplishments.

**Figure 1:**
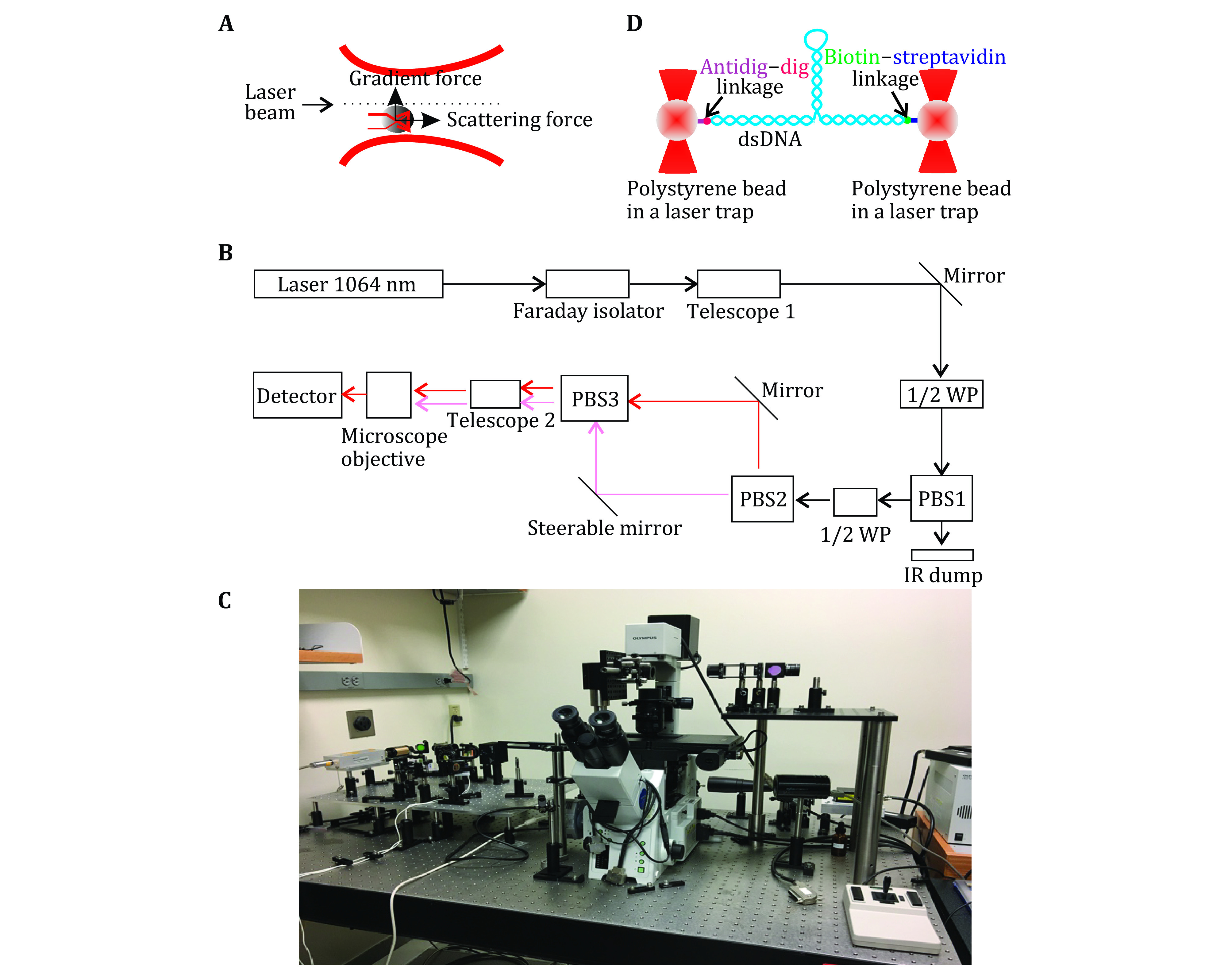
Optical tweezers principle and setup. **A** Single light beams gradient trap. **B** A dual trap optical tweezers diagram. **C** House-made optical tweezers set up in Dr. Guo’s lab at the Ohio State University (Ghimire *et al*. 2020). **D** Schematic showing a biomolecule tethered in a dual trap optical tweezers

**Figure 2:**
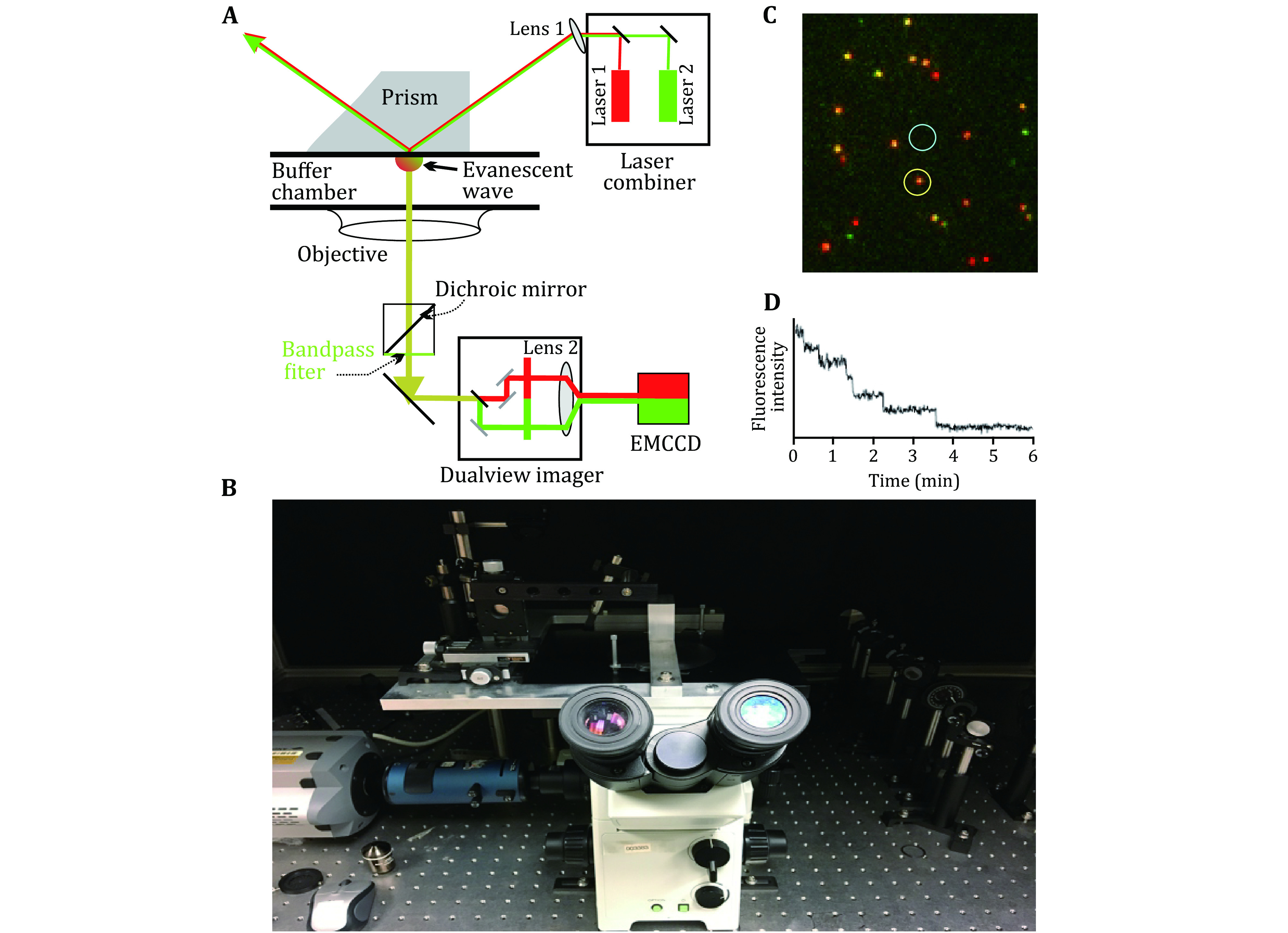
Total internal reflection fluorescence setup for single molecule fluorescence microscopy. **A** Prism based TIRF schematic diagram. **B** Prism based TIRF set up in Dr. Guo lab at the Ohio State University (Shu *et al*. 2007; Zhang *et al*. 2007, 2010a, 2010b). **C** Imaging of RNA dimers composed of one Cy3-RNA (green) and one Cy5 RNA (red). Yellow color indicates the overlap of Cy3 and Cy3, suggesting the formation of dimers containing both Cy3 and Cy5. **D** A typical fluorescence image of individual phi29 DNA packaging motor, showing six photobleaching steps, indicating that there are six copies of pRNA molecules on the DNA packaging motor

**Figure 3:**
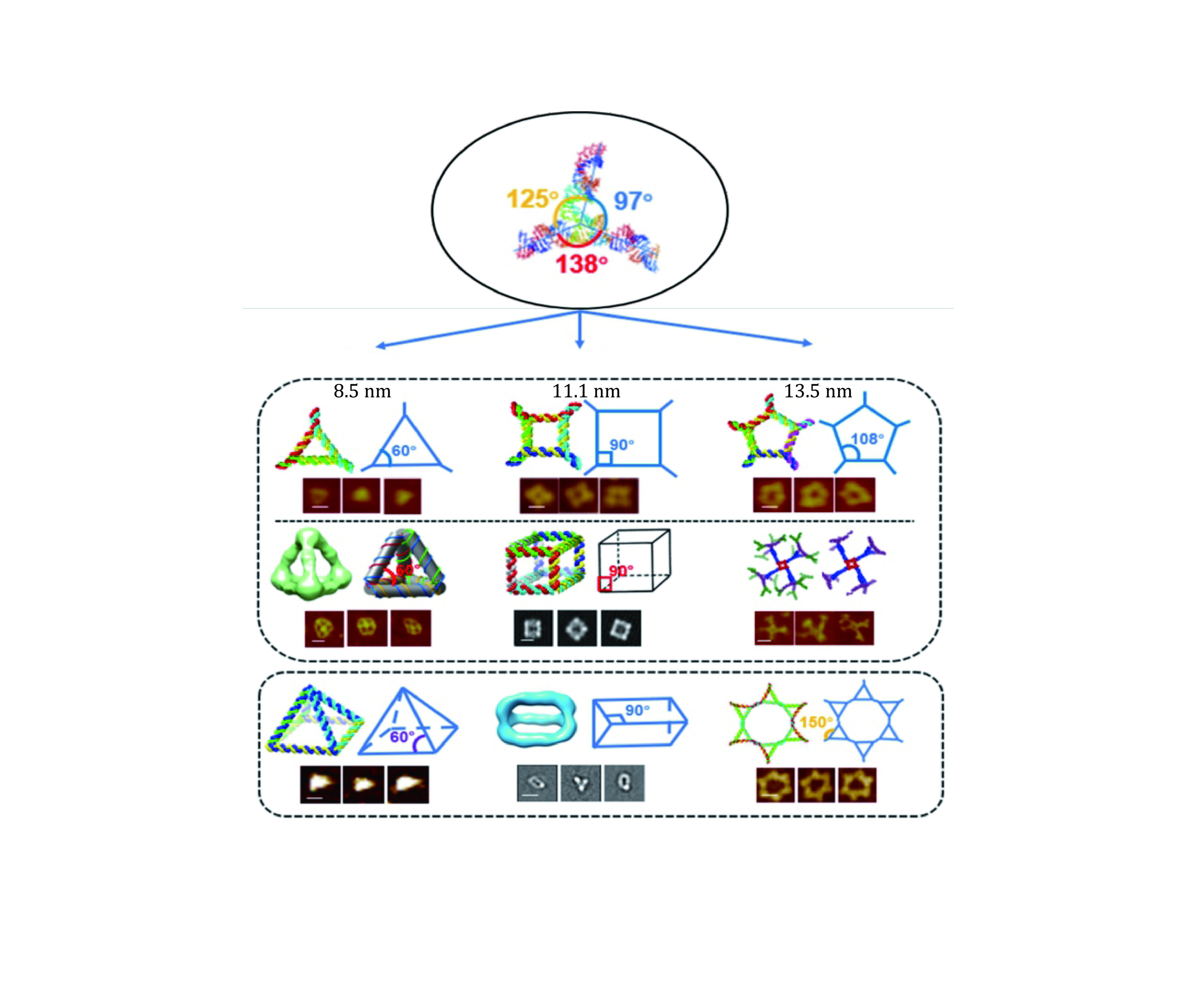
Construction and characterization of different RNA nanoparticles based on 3WJ. Angles of original 3WJ were stretched to accommodate different shapes of 2D or 3D RNA nanoparticles. In each of the structure, schematic and AFM/Cryo-EM images are shown. The 97° interior angle of the 3WJ was either compressed to 60° (left column), or stretched to 90° (center column) and 108° (right column), proving the rubbery property of RNA nanostructure. The scale bar is 10 nm in each image. Adapted from Ghimire *et al*. (2020) with permission from American Chemical Society

The most common optical instrumentation used in single-molecule studies is optical tweezers and smTIRF as shown in Fig. 1 and Fig. 2. Optical tweezers are the force-based technique that has revealed many molecular mechanisms as well as physical properties of biomolecules (Ghimire *et al*. 2020; Gordon *et al*. 2004; Gross *et al*. 2010). The analysis of RNA using optical tweezers has led to the discovery of the rubbery or amoeba property of RNA nanoparticles for compelling vessel extravasation to enhance tumor targeting and for fast renal excretion, thus solving two previous puzzles: (1) Why RNA nanoparticles can target tumor passively and efficiently? (2) why RNA nanoparticles remain non-toxic since they can be rapidly cleared from the body via renal excretion into the urine with little accumulation in the healthy organs in the body? These questions can be answered by the rubber- and amoeba-like deformation property that enables RNA nanoparticles to squeeze themselves out of the newly produced leaky blood vessel under the blood pressure. The elucidation of the rubbery mechanisms leads to the belief of RNA as less of nontoxic materials for cancer therapy. Various nanoparticles of different shapes and sizes have been constructed for this purpose as shown in Fig. 3. Single molecule photobleaching by smTIRF allows for the direct counting of biomolecules in a straightforward manner. This technique has been widely used for single molecule counting of RNA in phi29 DNA packaging motor (Guo *et al*. 2016). The technology has subsequently extended to the counting of the number of subunits or components in biological machines composed of protein, DNA, and other macromolecules. RNA, protein, or DNA molecules in nanoparticles can be counted, by combining it with single fluorophore labeling and statistical analysis. Single molecule photobleaching technology will provide insights into the RNA stoichiometry in nanoparticles, which is essential to a better understanding of various RNA functions, as well as better characterizations of RNA nanoparticles for nanomedicine. With better precision, sample preparation, and computation will allow these single-molecule methods to reveal biomolecular mechanisms in detail as well as may help to develop ultra-sensitive sensors to detect biomarkers and chemical species to be used in diagnostic and forensic. This review focus on the application of optical tweezers and smTIRF to study RNA and DNA nanostructure.

## OPTICAL TWEEZERS

### General principle of optical tweezers

Research in the life sciences has long been highly focused on the microscopic level, for their efficient and versatile functions in catching and controlling microspheres. Optical tweezers are recognized as a unique technique for research into single cells of living specimens, cell organs, and even biological macromolecules. Ashkin, the inventor of optical tweezers, predicted: “The ability to move organelles from their normal positions opens doors to sophisticated studies of cell function” (Ashkin 1986). The developments that have occurred during the past two decades have indicated that the technology of optical tweezers in elucidating basic life science questions has already facilitated many groundbreaking achievements and has by far surpassed the initial prediction of Ashkin in its actual contributions.

The photon has momentum, and this momentum will be imparted to the sample when the photon collides with it. For example, when a photon is absorbed or scattered by the object in question, the momentum of the photon will change. The object’s momentum will be also changed by a corresponding amount there must exist the conservation of momentum between the photon and the object. The change of the object’s momentum per unit time represents the total force of the object under illumination. The force exerted on the object by the radiation of the rays is generally termed the optical radiation pressure, or more simply optical pressure.

When light and matter interact with each other, there are two forces (scattering force and gradient force) acting on the matter. The scattering force is proportional to the intensity of the radiation along the direction of light beam propagation. The gradient force is proportional to the gradient of light intensity along the direction of the light intensity gradient. Typically, the force exerted on the object when radiation affects the object is a scattering force or a thrust force. However, as is shown in Fig. 1A, when the light field of a strong gradient (such as a laser beam) passes through the transparent particle, the gradient force may be larger than the scattering force.

The strong gradient light beam exerting energy on the particle forms a three-dimensional potential well. Once a particle crosses the potential barrier, it will be captured to the bottom of the potential well, *i.e*., it becomes trapped by the optical tweezers. The particle with the lowest potential energy will move with the movement of the optical tweezers so that objects can be manipulated in this way.

The size range of the particles that can be trapped and manipulated by optical tweezers varies from several dozens of nanometers to several dozen microns. The advantage of this large range is that the cells themselves and the cellular organelles are within this range (Zhong *et al*. 2013). Thus, its noninvasive attributes combined with the ideal range of experimental capabilities make it a logical system used by life scientists. Nanoscale particles can be adapted to allow biological components to be adhered to the particles, thereby providing a system that mirrors a manufacturing line where the products are nanoscale in size and the result being a complex of that size. When adopting a method of controlling particles by optical tweezers, the biological member is subjected to an external force, and the force will affect the particles. By measuring particles off the center of optical tweezers, the external force acting on the biological sample can be determined. This method has become a powerful tool to examine biological specimens.

Most optical tweezers experiments involve a tightly focused laser beam trapping a microscopic particle through optical gradient forces in all three dimensions. One potential drawback of these optical traps is that they can be relatively stiff, with a lateral spring constant of 100 pN/microgram, which exceeds the stiffness of most biomolecules studied with these instruments such as viral DNA molecules under moderate tension or single titin molecules. Therefore, measurements with such a stationary trap effectively impose constant-extension conditions upon the molecule, thereby providing conditions for experiments where force is measured as a function of extension (Bustamante *et al*. 1994).

For many applications, it is desirable to have a mode in which the molecules are maintained with a constant force regardless of their extension. For example, studies of motor proteins that move along the DNA strand, such as RNA polymerases or phage-head packaging motors (Keller *et al*. 2018). Similarly, conventional optical tweezers can be operated in a constant-force mode by using a feedback mechanism, which tracks the motion of the trapped particle by moving the focus of the laser beam or the distant attachment point of the molecule in such a fashion that the average displacement of the particle from the center of the trap remains constant. This type of feedback is rather slow and only provides average constant-force conditions but cannot respond quickly to fast individual events, such as a single step of a motor molecule. For experiments in a low-force or even zero-force regime or experiments with a high temporal resolution, another method is required (Jagannathan and Marqusee 2013). Detailed information on using optical tweezers to study various biomotors can be found in previous publications (Clemen *et al*. 2005; Knight *et al*. 2005; Maier 2005; Purohit *et al*. 2003).

Scanning-line optical tweezers with an asymmetric beam profile in the backplane of the microscope objective can be used to apply small, approximately constant, lateral radiation pressure to a trapped particle at a distance of several microns, thus eliminating the need for feedback mechanisms (Nambiar *et al*. 2004).

Optical tweezer technology has expanded the research of individual molecular behavior from the domain of "speculation" to true and feasible "experimentation", while realizing the possibility of controllable operations in nanoscale life processes. The optical tweezers control particles in a similar way to Hookean springs, in that they can survey tiny interactions in a real-time operating process. The tweezers are not only micro-mechanical manipulators but also small force microprobes. This characteristic has especially vital significance to biological macro-molecular behavioral research. There are many publications (Liesener *et al*. 2000; Moffitt *et al*. 2008; Neuman *et al*. 2008; Nieminen *et al*. 2007; Novotny *et al*. 1997; Zhang *et al*. 2013) describing the detailed principle and setup for optical tweezers (Fig. 1B and 1C). Optical tweezers have been used to solve many bimolecular puzzles including DNA, RNA–protein, and DNA–protein interactions (Jülicher and Bruinsma 1998).

### Optical tweezer to elucidate the elasticity of RNA and DNA

**Figure 4:**
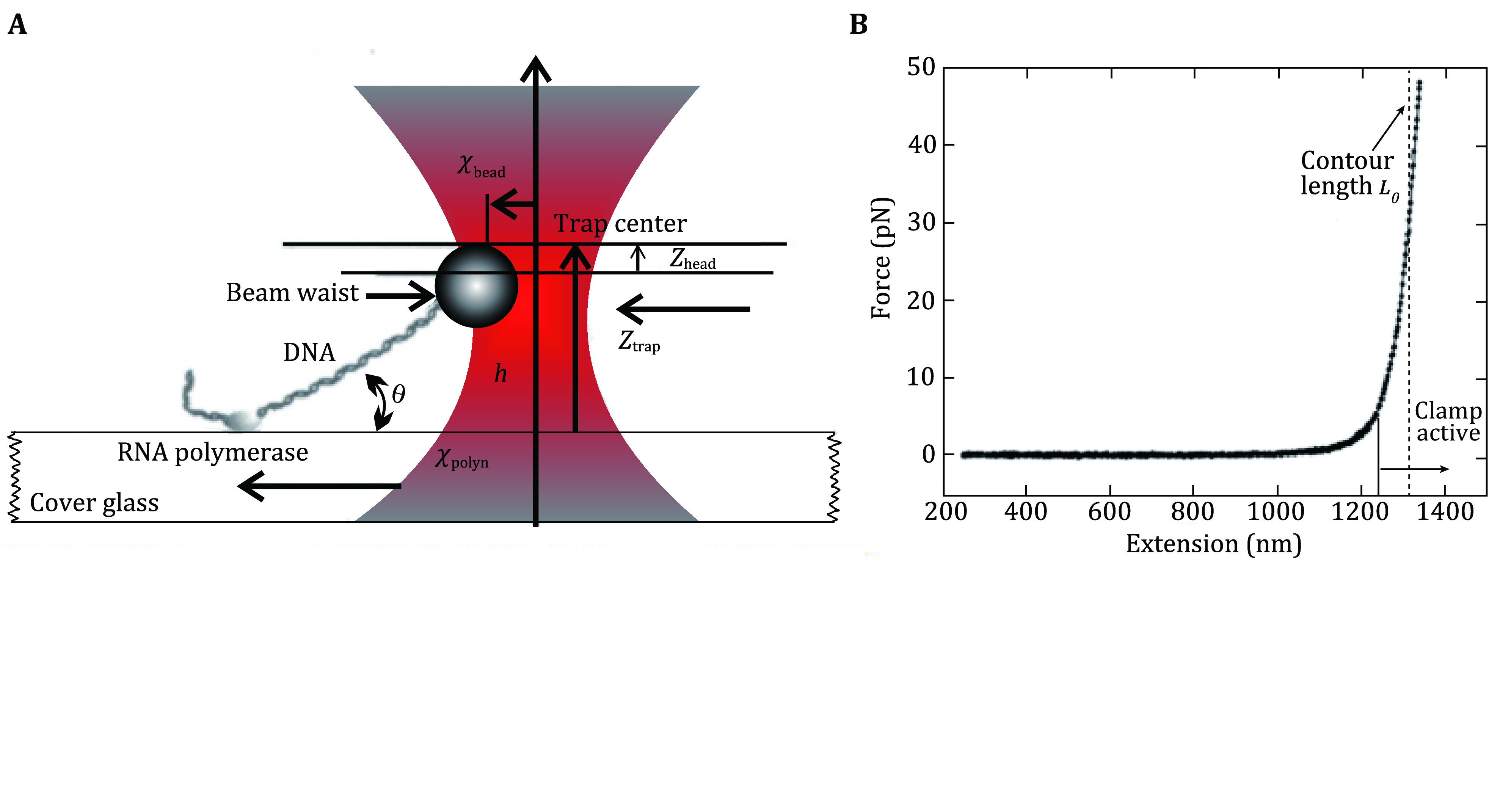
Stretching DNA with optical tweezers. **A** Experimental schematic showing DNA is anchored to the cover glass by a stalled RNA polymerase complex while the other end is attached to a bead held in the optical trap. **B** Force-extension curve of a single DNA molecule. Adapted from Wang *et al*. (1997) with permission from Elsevier

Elasticity refers to the stretchiness property of any material involving a change in shape upon application of force. It is one of the crucial properties of any material and it is highly desirable to understand the elasticity of biopolymers such as DNA, RNA and proteins evolved to serve lives. Optical tweezers have been an important tool to elucidate the elasticity of biopolymers since its discovery in 1986 (Ashkin 1986; Wang *et al*. 1997). Magnetic tweezers and AFM are also used to assay elastic properties of biopolymers at a single-molecule level (Abels *et al*. 2005; Zhang *et al*. 2019). An early research led by S.M. Block studied DNA elasticity using optical tweezers (Wang *et al*. 1997). In this study one end of a single DNA molecule was fixed on a glass surface using stalled RNA polymerase complex and another end was linked to a microscopic bead held in an optical trap (Fig. 4). The DNA was then stretched by moving the glass surface with respect to the bead using a piezo-driven stage while recording the position of the bead at nanometer resolution. This experiment yielded important information about the elastic behavior of DNA such as persistence length in various buffer conditions.

### Optical tweezers to quantify the forces in base paring

**Figure 5:**
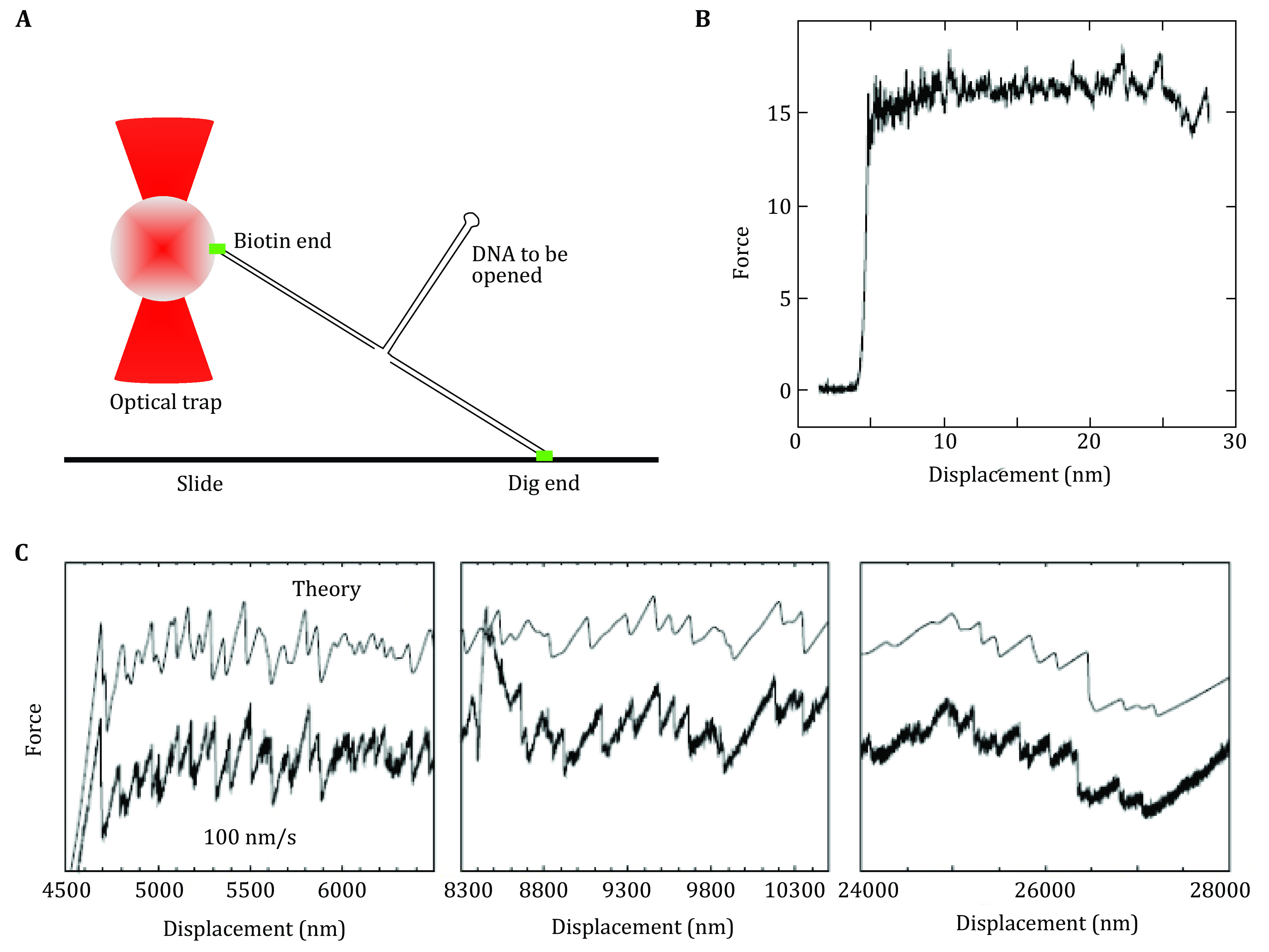
Unzipping DNA with optical tweezers. **A** Schematic of unzipping of DNA experiment. **B** Force versus extension curves corresponding to mechanical unzipping of a single lambda-phage DNA molecule. **C** The measured (bottom) and the calculated (top) curve for force vs extension curve for DNA. Adapted from Bockelmann *et al*. (2002) with permission from Elsevier

Unzipping experiments with DNA and RNA can measure the force required to break the hydrogen bonding between the base pairs of DNA and RNA. Bockelmann *et al*. studied unzipping of lambda DNA (Fig. 5A) using a molecular construct designed to unzip the DNA (Bockelmann *et al*. 2002). They studied the unzipping with 10 base pair sensitivity and found the unzipping force exhibits characteristic flips between different values at specific positions that are determined by the base sequence (Fig. 5B). This force flips causally related to the transitions between different states involved in the time-averaging of the molecular system. The authors compared the forced unzipping results with the theoretical calculations and found them in fair agreement with some variation along the force curve (Fig. 5C).

### Optical tweezers to study unfolding of RNA nanostructures

The nanomanipulation capability of optical tweezers has been utilized to monitor the unfolding/folding of individual structural domains such that the hierarchical folding of a large RNA. Alternatively, a single strand can be directed to fold into one of several conformers; or an existing structure can be mechanically induced to refold into a different conformation. Under biological conditions, RNA structure can be altered upon temperature change, ligand binding including induced fit. The capability of directly manipulating molecular structure opens a new venue for RNA structural study.

**Figure 6:**
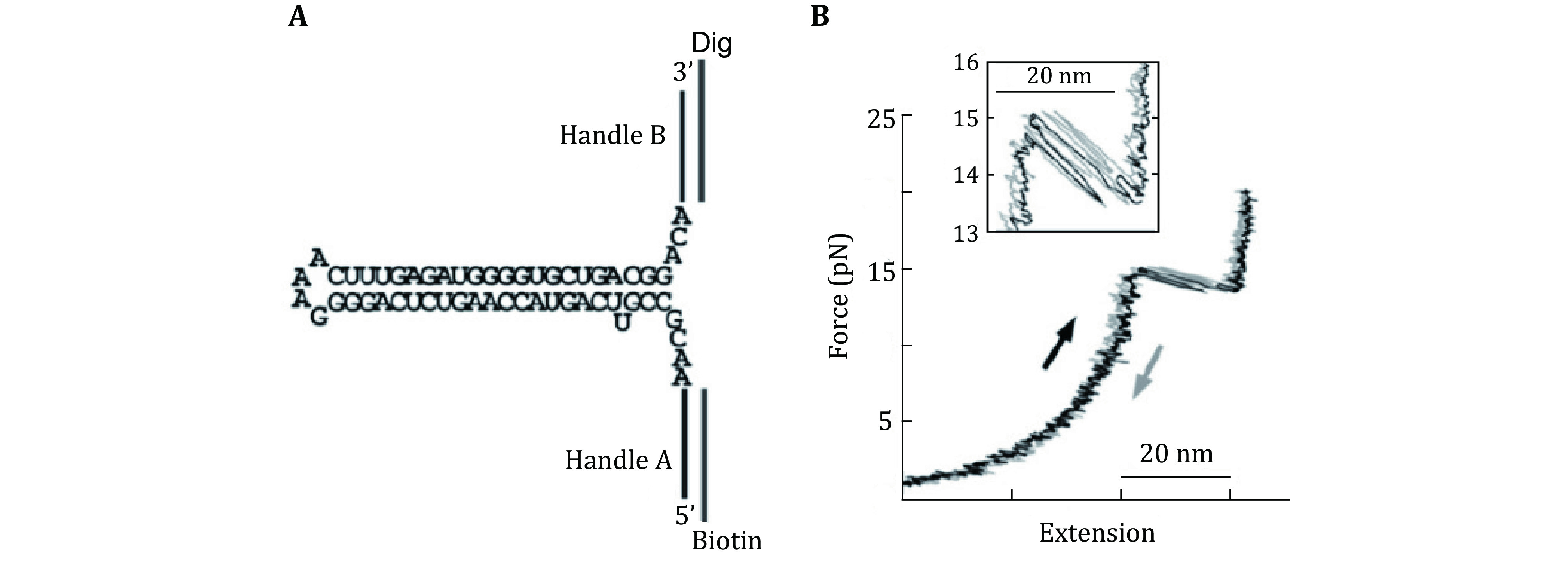
Unfolding of RNA structure. **A** RNA construct flanked by DNA handles for unfolding experiment by optical tweezers. **B** Force vs extension curve of the RNA construct stretching (black) and relaxing (shaded). Adapted from Wen *et al*. (2007) with permission from Elsevier

Wen *et al*. applied optical tweezers to determine experimental variables of optical tweezers instrumentation that affect RNA folding/unfolding kinetics (Wen *et al*. 2007). They used hairpin structure flanked by hybrid RNA/DNA handles tagged with biotin and digoxigenin molecules at the ends to bind with polystyrene beads coated with streptavidin and anti-digoxigenin antibody, respectively (Fig. 6). They found that the stem-loop size and base composition affects the folding/unfolding kinetics more than the experimental parameters. Stephenson *et al*. used the nanomanipulation capability of optical tweezers to study the folding of structural domains in RNA secondary and tertiary structures (Stephenson *et al*. 2014). Che *et al*. used optical tweezers to unfold RNA pseudo-knots and discovered the two-stage unfolded state, and even detected transient experimental evidence between the two folded states aiding in the explanation of the complex path of structural transitions in biomolecules (Manosas *et al*. 2007). Whitley *et al*. discovered the elastic properties of nucleic acid hybridization transition states. They found that the properties are like pure single-stranded states. Le *et al*. used biochemical, genetic, optical tweezers combined with coarse horizontal and fine particle (SMD) methods to study the T-shaped RNA structure during the decomposition of the radish wrinkle virus (Le *et al*. 2017). They found a complex biologic switch and folding/unfolding dynamics of other complex RNA structures.

### Optical tweezers to study unfolding of DNA nanostructures

**Figure 7:**
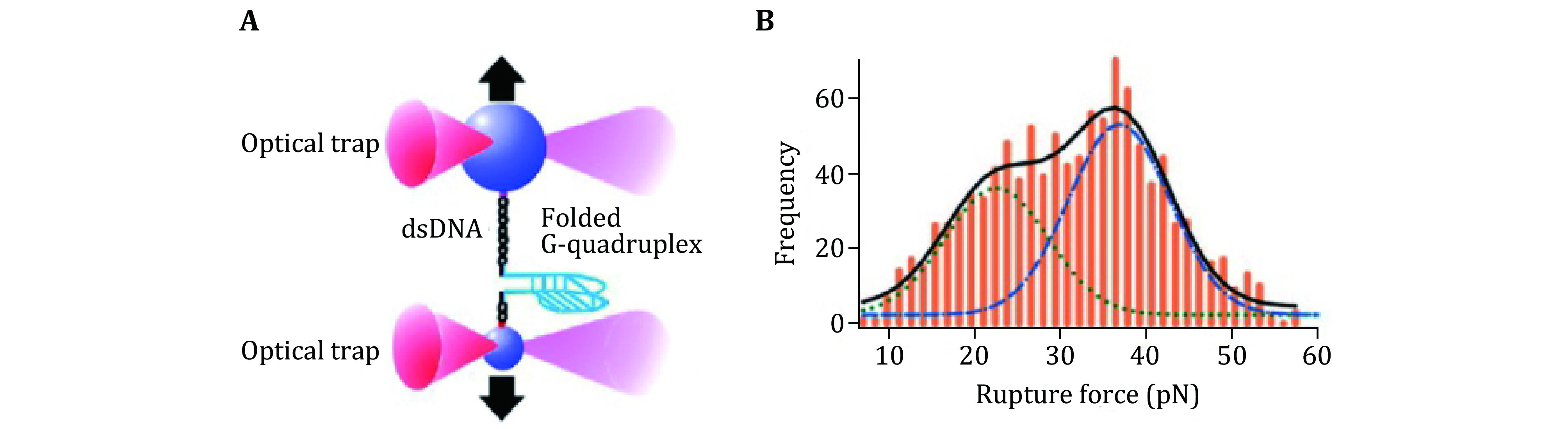
Unfolding of DNA G-quadruplex with optical tweezers. **A** Schematic of experimental design. **B** Rupture force histogram for the unfolding events. Adapted from Yu *et al*. (2009) with permission from American Chemical Society

In a study led by Hanbin Mao, dual trap optical tweezers were used to study the nanostructure of intramolecularly folded G-rich DNA sequence called G-quadruplex (Yu *et al*. 2009). Yu *et al*. found that G-quadruplexes formed in the G-rich regions have high mechanical stability and unfolding forces are higher than the stall forces of enzymes having helicase activities as shown in Fig. 7. In another study of G-quadruplex, Koirala *et al*. used optical tweezers as a platform to assay interactions between G-quadruplexes and small-molecule ligands (Koirala *et al*. 2011). The authors mentioned that this single-molecule method can be used to profile the mechanical, kinetic, and thermodynamic properties of ligand biomacro-molecules interaction. This strategy could be developed into a sensitive drug screening method. Ghimire *et al*. dissected the G-quadruplex through different planes and found that mechanically loop interaction contributed more to the G-quadruplex stability than the stacking of G-quartets (Ghimire *et al*. 2014). In a similar study they also investigated mechanical anisotropy of ligand–biomolecule complexes (Jonchhe *et al*. 2019). The researchers used optical tweezers to dissect the G-quadruplex through different planes and loops with and without telomestatin derivative, a G-quadruplex binding small molecule. They found that the ligand binding in the core region of the G-quadruplex increases the mechanical stability of the core region, but it weakened stability in the loop region. Shrestha *et al*. used optical tweezers to study DNA origami comprising of various shapes such as cubes, pyramids, and tiles (Shrestha *et al*. 2016). They studied the mechanical stability of DNA origami structures and the mechanical isomerization. Particularly, they observed two conformations of DNA nanotubes at 10–35 pN. Their results demonstrated that the Holliday junctions’ control the mechanical behaviors of DNA architectures in the DNA origami.

### Optical tweezer to elucidate the elasticity of RNA nanoparticles

**Figure 8:**
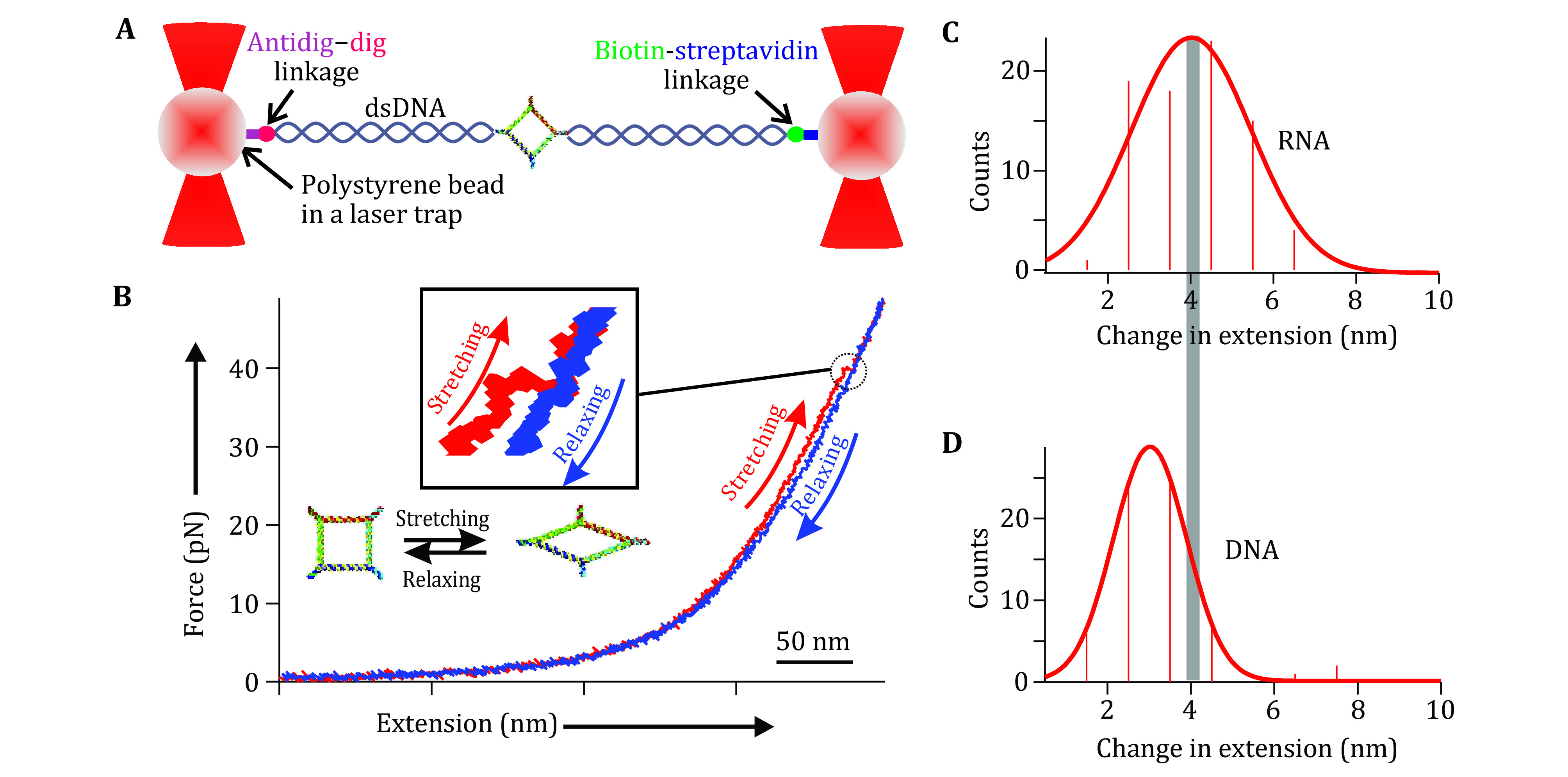
Rubbery property of RNA square as demonstrated by optical tweezer analysis. **A** Schematic diagram of dual trap optical tweezers with a tethered RNA construct sandwiched between two dsDNA handles via affinity linkers. **B** A typical force-extension curve for stretching (red) and relaxing (blue) of square nanoparticle. Inset shows magnified view of conformational change of the square nanoparticle. The 50 nm scale bar is for the *x*-axis of force extension curve. Adapted from Ghimire *et al*. (2020) with permission from American Chemical Society

The team led by Peixuan Guo developed a method to understand the rubbery property of RNA nanomaterials by force and extension measurements with dual beam optical tweezers (Ghimire *et al*. 2020). RNA nanoparticles were prepared by mixing five strands at a stoichiometric ratio to self-assemble. Phi29 3WJ sequences incorporation at each corner made the 2D nanosquare assembled with high thermodynamic stability. Two diagonal overhangs were created for dsDNA handle ligation to enable the optical tweezer measurement. The square RNA was connected to two dsDNA handles by ligation to attach to the polystyrene beads at the ends through a biotin-streptavidin and dig-antidigoxigenin linkage separately (Fig. 8A). Force ramping experiments were performed along the vertices of each square, using a loading rate of 5.5 pN/s by moving one of the trapped particles away from the other using a steerable mirror to gradually increase the force (Fig. 8A). A sudden jump in force-extension curves recorded during the experiments confirmed a change in conformation of the square (Fig. 8B). The RNA conformational change reverted to the original conformation slowly as shown in the relaxing curve (Fig. 8B) after lowering the force. This reversible feature of the conformational change of the RNA nano-square demonstrated the rubbery property of RNA nanosquares.

A research team led by Peixuan Guo quantified the stretchiness of the particles from the force-extension curves obtained from the force ramping experiments. A change in extension at a given force was obtained as a difference between the extension of stretching and relaxing curves (Fig. 8C, 8D). It was found that among the RNA and DNA squares, RNA squares can extend 4.1 ± 0.10 nm under stretching force, while the DNA square can extend 2.99 ± 0.03 nm only. It was found that among the RNA and DNA squares, the change in extension for RNA is longer at 4.1 ± 0.10 nm, compared to 2.99 ± 0.03 nm for DNA. It is well known that RNA and DNA exhibit differences in their elasticity due to their respective A- and B-form helical structures. Their Young’s moduli are well-studied by determining the extension and releasing force measured by force spectroscopy (Clemen *et al*. 2005). This difference could be due to the natural difference between RNA and DNA configuration. The A-form and B-form is different concerning the structure of helix and a different number of nucleotides per helical turn (Clemen *et al*. 2005; Churchman *et al*. 2005; Michelotti *et al*. 2010). The dsRNA generally displays an A-form with 11 bases per helix tun, while the dsDNA generally displays a B-form with 10.5 bases per helix tun. This difference specifically may have been contributed by the C3’-endo and C2’-endo conformation of the A-form and B-form RNA and DNA, respectively. The C3’-endo form of RNA has a phosphate-to-phosphate distance of 0.59 nm while that for the C2’-endo form on DNA is 0.7 nm. Thus, Guo group speculated the compact structure of the RNA may have contributed to the length change in extension for the nanosquare made of dsRNA. This result demonstrates the highly flexible and more rubbery behavior of the RNA nanoarchitecture compared to its DNA counterpart.

A histogram of force required to change the conformation obtained from the force-extension curve depicted the mechanical stability of the particle. The plot of force shows high mechanical stability of the RNA, and DNA squares, at nearly 45 pN (Table 1). Having a similar force profile but a different change in extension demonstrates the more stretchable property of RNA nanosquare under similar stress. Kinetics of change in conformation was analyzed by fitting the force histograms of each square to an equation proposed by Dudko *et al*. (Dudko *et al*. 2008). Results show that the change in activation energy is smallest for RNA as shown in Table 1. RNA also has a faster change in conformation, as demonstrated by its conformation change rate constant, and the smallest distance to the transition state from the state prior to the change in conformation. The lower activation energy required to change the conformation of RNA may explain the more flexible nature of the RNA nano-squares under tension.

**Table 1:** Extension change (∆*x*) corresponding to force (Force)

	Force (pN)	∆*x* (nm)	*k*_cc_ (s^−1^)	*x*^†^ (nm)	∆*G*^†^ (kcal/mol)
DNA	44.2 ± 2.3	2.99 ± 0.03	0.0016 ± 0.0007	0.132 ± 0.008	13.5 ± 0.5
RNA	44.6 ± 4.1	4.1 ± 0.10	0.005 ± 0.0030	0.10 ± 0.020	11.4 ± 1.4
The value for DNA and RNA concerning conformation change rate constant (*k*_cc_), distance to the transition state (*x*^†^) and conformation change energy barrier (∆*G*^†^) are shown. Adapted from Ghimire *et al*. (2020) with permission from American Chemical Society					

### The optical tweezer elucidated rubber property of RNA nanoparticles supported by *in vivo* imaging technology

A Research led by Peixuan Guo group had previously demonstrated that, based on high thermodynamic stability, controllable size, shape, and stoichiometry, RNA nanoparticles can serve as therapeutics delivery vehicles loaded with chemical drugs and RNA therapeutic agents for tumor targeting even without the tumor binding ligands (Ouyang *et al*. 2020). The strong enhanced permeability and retention (EPR) effect of RNA nanoparticles has been extensively demonstrated as a drug delivery platform (Annibale *et al*. 2011; Lillemeier *et al*. 2010; Rajoo *et al*. 2018). The appropriate size of RNA nanoparticles results in a strong EPR effect while avoiding entrapment in the liver and spleen by the mononuclear phagocytic system (MPS) recognition (Ha and Tinnefeld 2012; Leake *et al*. 2006; Sengupta *et al*. 2013). Systemically injected RNA nanoparticles strongly and specifically bind to cancers with reduced accumulation in the liver, lung, or any other vital organs or tissues (Arumugam *et al*. 2009; Lillemeier *et al*. 2010; Simonson *et al*. 2010; Ulbrich and Isacoff 2007). Some other targeted theraupetics have also been reported (He *et al*. 2020; Liu *et al*. 2020; Ouyang *et al*. 2020).

**Figure 9:**
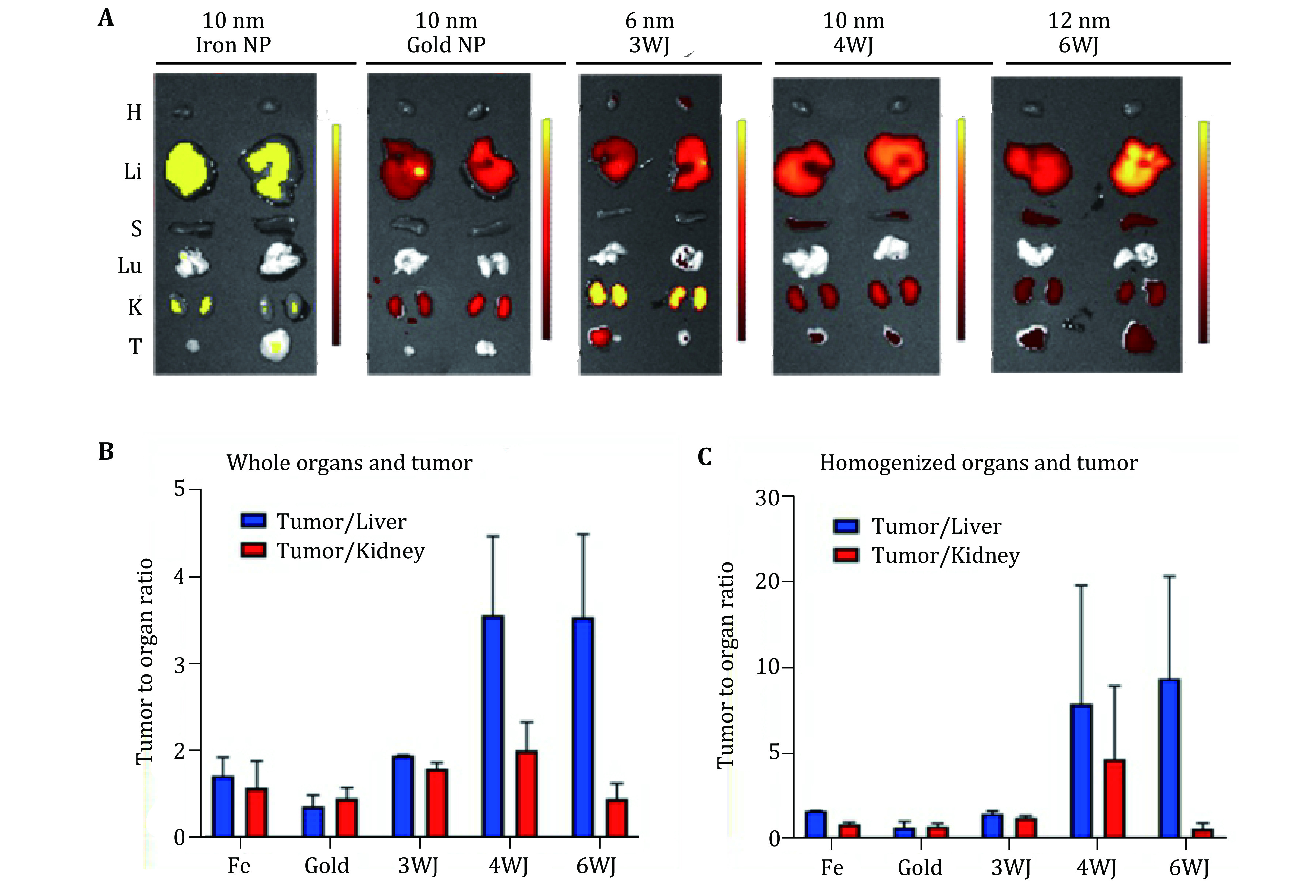
Demonstration of the rubbery property of RNA nanoparticles by comparing the retention time in tumor, kidney, and liver. **A** The Cy5.5 labeled nanoparticles were detected by *ex vivo* organ 8 h post-injection in mice bearing KB xenograft (T: tumor, H: heart, S: spleen, Lu: lung, K: kidney, and Li: liver; Color scale: radiant efficiency (p⋅s^−1^⋅cm^−2^⋅sr^−1^) / (μW⋅cm^−2^)). **B** Quantitative analysis of whole body biodistribution to quantify the ratio of tumor to liver and tumor to kidney using images from **A**. **C** Quantitative analysis of biodistribution in tumor to liver and tumor to kidney ratio, quantified from the homogenized organ sample. Adapted from Ghimire *et al*. (2020) with permission from American Chemical Society

It is expected that the phenomenon of tumor accumulation without the use of targeting ligands is due to the EPR effect which leads to the accumulation of RNA nanoparticles in the cancer vasculature without cell entry (Prabhakar *et al*. 2013). To further investigate the mechanism for such favorable accumulation in the cancer vasculature, we quantitatively compared tumor uptake, organ retention, and kidney clearance of RNA nanoparticles to solid inorganic nanostructures, such as iron, and gold of similar or same size (Fig. 9). To compare the EPR effect and the proposed rubbery property of RNA nanoparticles, NIR fluorescent dye labeled 6 nm RNA 3WJs, 10 nm 4WJs, 12 nm 6WJs, 10 nm gold nanoparticles, and 10 nm iron nanoparticles were injected intravenously into mice. The *in vivo* organ distributions of various RNA nanoparticles and their inorganic nanoparticles counterpart were examined 8 h post injection. The fluorescent intensity of *ex vivo* entire organ as well as homogenized organ sample were quantified. It was found that at 8 h, RNA nanoparticles of different stoichiometry and the iron or gold nanoparticles exhibit big differences in organ and tumor retention (Fig. 9A). After normalizing the fluorescence efficiency by organ weight, 4WJ-RNA nanoparticles possess a higher tumor to liver and tumor to kidney ratio (Fig. 9B). The homogenized organ sample data are consistent with the *ex vivo* result (Fig. 9C), which further demonstrates the favorable biodistribution profile of RNA nanostructures. Interestingly, the tumor to organ fluorescence ratio, of the 10 nm RNA nanoparticles was much higher than the iron and gold with the same size of 10 nm. These results support the hypothesis that RNA nanoparticles display rubbery property, leading to the stronger EPR effect and enhanced vessel extravasation in tumor targeting supporting the rubbery behavior evaluated in the single molecule optical tweezers experiments.

Chemicals or nanoparticles are excreted mainly by renal excretion. In this process, these small complexes are filtrated via the kidney into the bladder and ultimately into the urine. The rubbery property of RNA nanoparticles was also demonstrated by injecting different nanoparticles intravenously into the mice, and the distribution and clearance of the nanoparticles were monitored by whole body imaging. Larger nanoparticles were circulated longer with final clearance by renal excretion from the body. Even the 20 nm squares were removed from the renal system which has filtration cut off < 5.5 nm. These results suggested the flexible nature of the nanoparticles may allow them to pass through the kidney due to the rubbery property of RNA nanoparticles. This deformation property of RNA nanoparticles enables it to squeeze through leaky vessels of the tumor vasculature improving the EPR effect allows the intact RNA nanoparticles to squeeze through renal filters for urine excretion without toxicity.

**Figure 10:**
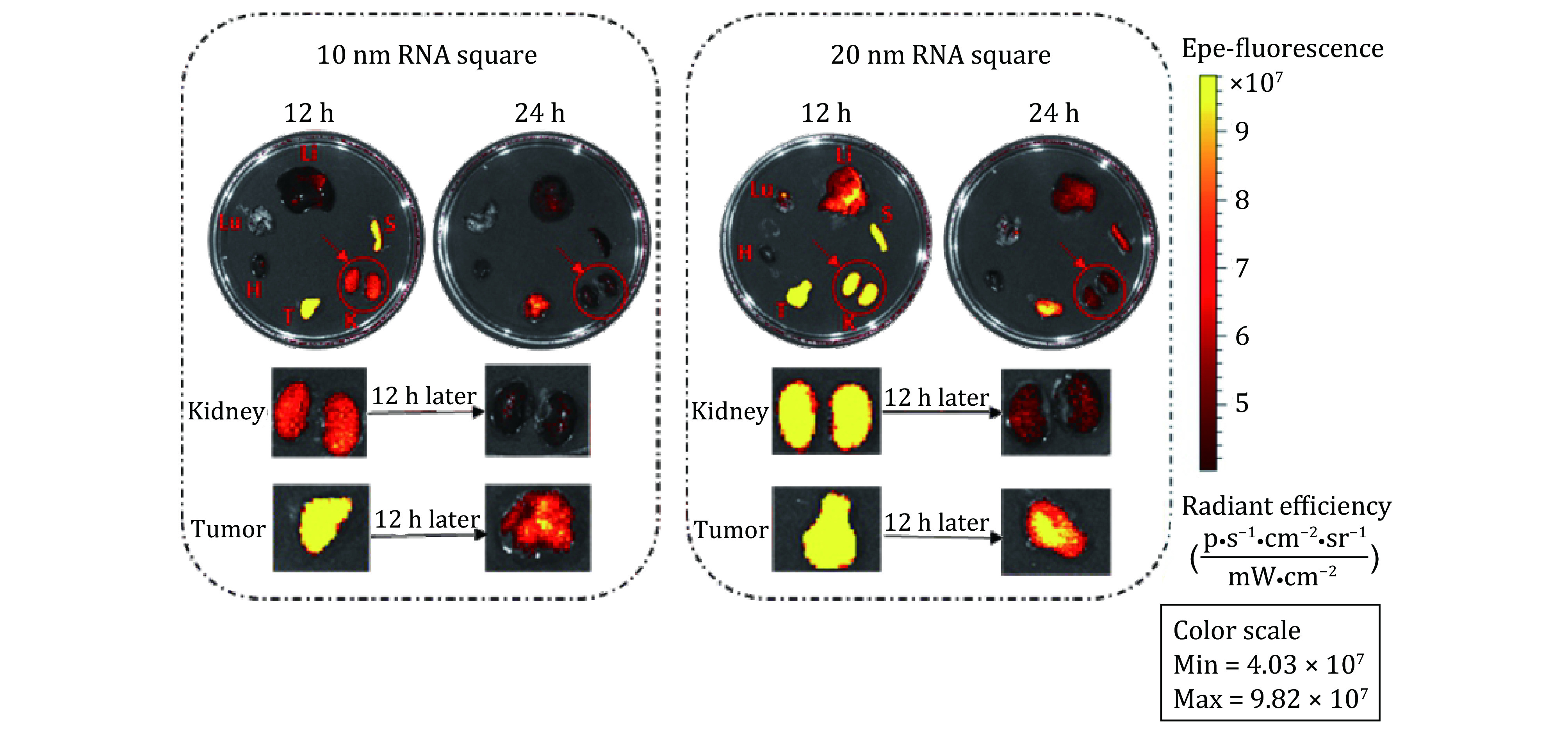
Demonstration of the rubbery property of RNA nanoparticles through renal excretion by comparing tumor and kidney retention time using *ex vivo* organ images. The near-infrared AF647 labelled 10 nm and 20 nm squares were detected in the kidney 12 and 24 h post systemic injection. Both the 10 and 20 nm RNA squares were excreted through kidney filtration, while with longer retention time in tumor by comparing the organ intensity after 12 h. Adapted from Ghimire *et al*. (2020) with permission from American Chemical Society

The biodistribution of RNA nanoparticles is size and shape dependent as reported previously and timely clear out from the organs (Fig. 4 in Jasinski 2018). In another recent work, mice were sacrificed, and organ samples were collected and imaged for *ex vivo* organ fluorescence at time points of 12 h (left) and 24 h (right), respectively (Fig. 10). *Ex vivo* organ images of 10 and 20 nm RNA squares suggested that the RNA nanoparticles larger than the 5.5 nm renal execration limit passed the kidney filtration. As evidenced, near-infrared AF647 labeled 10 and 20 nm squares were detected both in kidney and tumor 12 and 24 h post injection; however, the kidney signal significantly reduced after 12 additional hours whereas the fluorescence in tumor had a minor reduction. These large RNA squares were excreted through kidney filtration and they also stayed in the tumor with a longer retention time in comparison to that in the kidney. Since the upper limit of the renal excretion is about 5.5 nm, the renal excretion and without body accumulation of the nonvolatile and non-degradable RNA nanoparticles suggests their rubbery property.

**Figure 11:**
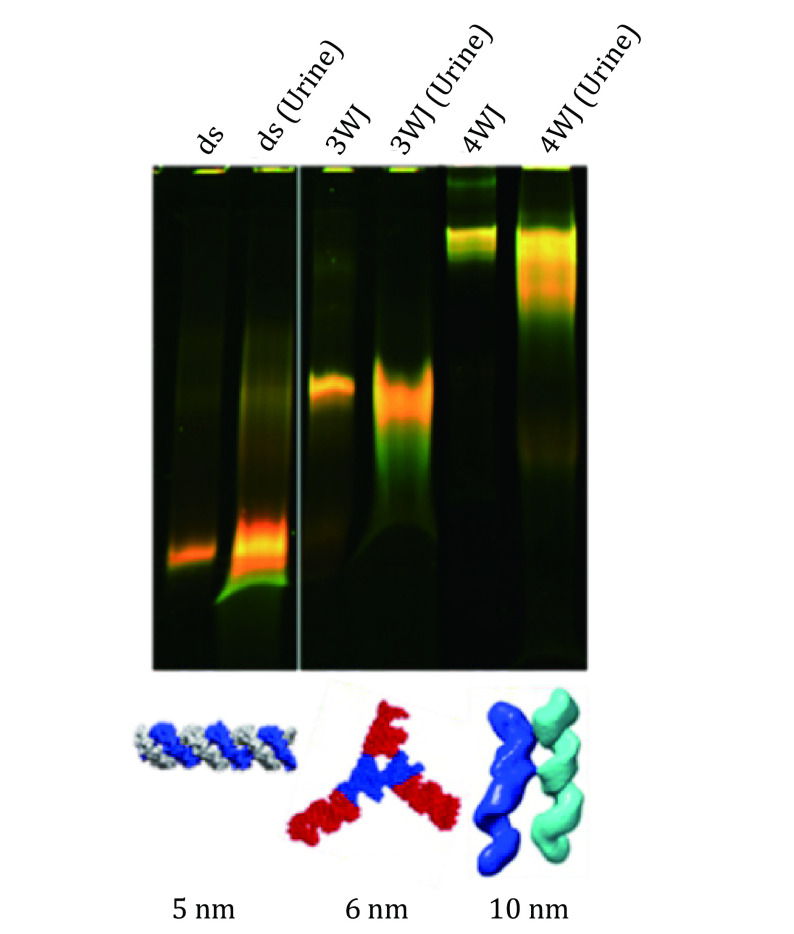
Demonstration of the rubbery property of dsRNA and RNA nanoparticles in urine sample after 30 min of IV injection. Near-infrared AF647 labelled dsRNA, RNA 3WJ and 4WJ were found in mice urine assayed by 12% native gel 0.5 h post IV injection. Adapted from Ghimire *et al*. (2020) with permission from American Chemical Society

To further confirm the rubbery property of RNA nanoparticles during renal excretion, Ghimire *et al*. group published a work in which we injected the nanoparticles of different sizes and collected mice urine samples for further investigation (Ghimire *et al*. 2020). In this study, double strand(ds-), 3WJ- and 4WJ-RNA nanoparticles were injected into mice. Urine was collected 0.5 h post systematic injection and assayed by 12% polyacrylamide TBE (89 mmol/L Tris base, 200 mmol/L boric acid, and 2 mmol/L EDTA) gel electrophoresis. Intact 5, 6, and 10 nm RNA nanoparticles were detected in the gel from urine samples (Fig. 11), suggesting direct kidney filtration of RNA nanoparticles larger than the kidney filtration limit.

Direct glomerular filtration is highly dependent on molecule size and structure because the physiologic pore size is 4.5–5 nm after considering the combined effects of each layer of the glomerular capillary wall. Smaller molecules with a hydrodynamic diameter (HD) < 5.5 nm are filtered directly, while larger molecules cannot be eliminated by the glomerulus (Du *et al*. 2017). The 3WJ- and 4WJ-RNA nanoparticles are larger than the filtration slit. However, they were excreted very quickly by the direct kidney filtration as the complete nanoparticles shown in the gel (Fig. 11), suggesting that the RNA nanoparticles may tune their shape to fit and go through the filtration slit and recover back to its original structure like rubber after passing through the pore.

## SINGLE-MOLECULE TOTAL INTERNAL REFLECTION FLUORESCENCE (SmTIRF)

### General principle of smTIRF

Fluorescence based imaging is the most widely used technique to reveal the bimolecular process *in vitro* as well as *in vivo* (Lichtman and Conchello 2005). Extension of fluorescence methods at the single-molecule level beyond optical diffraction limit have provided information about several important biological species and process with great details otherwise would have remained elusive (Moerner and Fromm 2003; Klar *et al*. 2000). For detection with fluorescence measurements, reducing the background signal is significant to achieve better sensitivity and precision (Moerner *et al*. 2003). TIRF is a widely used fluorescence-related method to reduce background noise (Roy *et al*. 2008). In TIRF, the evanescent wave can only be produced when the incident light is totally internally reflected at the interface of the medium (Martin-Fernandez *et al*. 2013). The totally internal reflection can be achieved by controlling the incident light angles and the refractive index of different materials (Gibbs *et al*. 2018). Since the evanescent electromagnetic field decays exponentially from the interface, the light can only penetrate approximately 100 nm depths into the sample medium. Therefore, TIRF only visualizes the samples around the surface regions. Bulk samples outside the restricted areas could not be excited by the laser light to cause high background noises. The low background signal feature of TIRF makes it an excellent research tool for single-molecule detection (Kaur and Dhakal 2020). Abundant information of the samples, such as the motion of the molecules from the TIRF imaging, can be deduced. Moreover, along with the photobleaching measurements, the number of fluorophore-labeled molecules can be counted from the stepwise fluorescence intensity drop, which could provide the conformation of different molecules (Das *et al*. 2007; Gordon *et al*. 2004; van Dijk *et al*. 2004; Zhang and Guo 2014; Zijlstra *et al*. 2012). Presently, TIRF plays essential roles in various fields, especially in cell and molecular biology (Tokunaga *et al*. 1997).

However, many molecules in biological systems are densely packed; hence, high-resolution images are difficult to be obtained due to the diffraction limit, which is about 200 nm for visible light. Molecules that reside within this limit cannot be distinguished from each other by conventional optical microscopy. In recent years, many super resolution microscopy techniques have been developed, and biological samples can now be imaged with nanometer precision (Churchman *et al*. 2005; Gordon *et al*. 2004; Hess *et al*. 2009; Huang *et al*. 2008; Klar *et al*. 2000; Michelotti *et al*. 2010; Qu *et al*. 2004; Rust *et al*. 2006; Yildiz and Selvin 2005). In particular, the photoactivated localization microscopy (PALM) and stochastic optical reconstruction microscopy (STORM) are methods enabling imaging over the diffraction limit. Briefly, a set of non-overlapped fluorescence molecules are excited to take images and the fluorescent molecules will then undergo photobleaching or switch to a dark state. This process will be repeated with another set of fluorescence molecules. All the image or position data will be integrated to make images with super-resolution. Currently, PALM and STORM (Singh *et al*. 2007), based on localization of a stochastically photoactivated molecule have been applied for quantitative analysis of fluorescent molecules, providing information such as stoichiometry, size, and arrangement of the bio-complexes (Annibale *et al*. 2011; Lee *et al*. 2012; Lillemeier *et al*. 2010; Rajoo *et al*. 2018; Sengupta *et al*. 2013). However, some molecules may not be photoactivated during PALM imaging, making it difficult to determine the absolute number of molecules and leading to underestimation of the stoichiometry. The mechanism and potential challenges in stoichiometry quantification by PALM might also apply to STORM (Rust *et al*. 2006).

The stoichiometry of subunits in biomolecules is essential in cell and molecular biology. Single-molecule photobleaching for direct counting, developed by Guo’s lab in conjunction with single-molecule photobleaching, render more versatility on the choice of fluorophores, which does not require photoactive fluorescent molecules. Moreover, the utility of single-molecule photobleaching has become a simple and straightforward way to study stoichiometry of subunits in bio-complexes (Shu *et al*. 2007; Zhang *et al*. 2007). This technology has now been used extensively for single molecule counting of protein, RNA, and other macromolecules in a variety of bio-machines and nanostructures (Amrute-Nayak and Bullock 2012; Arumugam *et al*. 2009; Cherny *et al*. 2010; Coffman and Wu 2012; Das *et al*. 2007; Ding *et al*. 2009; Ha *et al*. 2012; Jiang *et al*. 2011; Leake *et al*. 2006; McGuire *et al*. 2012; Mehta *et al*. 2013; Revyakin *et al*. 2012; Shu *et al*. 2007; Simonson *et al*. 2010; Ulbrich *et al*. 2007; Yokota *et al*. 2013; Zhang *et al*. 2007; Zijlstra *et al*. 2012). Unlike PALM (Lee *et al*. 2012) and STORM (Huang *et al*. 2008), single-molecule photobleaching cannot provide information on size, dimension parameters, or spatial distribution of the molecules, but render more versatility on the choice of fluorophores, which does not require photoactive fluorescent molecules. Moreover, the mathematic algorithms are not necessary for single molecule photobleaching.

Details of instrumentation of single molecule total internal reflection fluorescence (TIRF), prism-type and objective-type have been published previously (Martin-Fernandez *et al*. 2013; Roy *et al*. 2008; Yildiz *et al*. 2003; Zhang *et al*. 2007, 2010a, 2010b, 2014). Briefly, there are two general types of the TIRF setup. One is prism type and the other is objective type. In prism-type TIRF, the sample perfusion chamber is usually comprised of a quartz slide on top and a glass coverslip below (Fig. 2). The contact between the bottom surface of the quartz prism and the top surface of the quartz slide of the chamber was mediated by oil having a refractive index close to that of quartz. The excitation laser beam was elevated through a series of optics to shine to the side of the quartz prism, which was fixed directly right above the objective’s field of view by a customized prism holder; the incident angle was adjusted so that after its refraction through the prism, the beam incited onto the quartz/water interface with an incident angle larger than the critical angle for total internal reflection of the beam. Objective-type TIRF is achieved by using a microscope objective with a numerical aperture (NA) larger than 1.38, and an angular aperture allowing an incident angle larger than the critical angle for total internal reflection at the glass/water interface. The excitation beam is expanded and then focused on the back focal plane of the objective through a lens next to the back aperture of the microscope. A dichroic mirror in the microscope filter cube is used to reflect the beam to the objective. The sample is excited by the laser beam through the TIRFM objective. By adjusting the position of the lens, the beam can be aligned to the side of the objective, and thus achieve total internal reflection through the objective lens. Fluorescence signals are detected with a back-illuminated electron-multiplied charge-coupled device (EMCCD) camera which is operated at −80 °C to reduce the thermal-dependent dark noise. A commercially available multiview imager is used to split fluorescence signals of different wavelengths before they reach the EMCCD camera. The optics are aligned so that the multiple signals reach the detector, and thus can be recorded simultaneously (Gordon *et al*. 2004; Knight *et al*. 2005). Various pairs of fluorophores can be simultaneously imaged and co-localized with different combinations of lasers in the laser combiner and filter sets in the imager.

In this section, we focus on the studies of TIRF in the RNA nanotechnology and the direct counting of RNA subunits within nanometer sized biological nanoparticles using the single molecule photobleaching (SMPB) approach.

### Single molecule photo bleaching technology in single molecule counting of RNA

A quantized intensity drop of photobleached single fluorophore helps to directly count the number of fluorophores within a diffraction limited fluorescent spot over time. This method has been used to determine the stoichiometry of packaging RNA (pRNA) in the bacteriophage phi29 DNA packaging motor (Shu *et al*. 2007; Zhang *et al*. 2010a). Further photobleaching studies on the phi29 DNA packaging motor facilitated the elucidation of a novel mechanism of RNA/protein interaction (Zhao *et al*. 2013) and to determine the copy number of viral RNAs packaged in a single influenza virus particle (Chou *et al*. 2012). This method can be applied to single molecule counting of RNA, DNA, protein, and other molecules in nanoparticles, macromolecule complex, and biological complexes. Here, we describe its application focusing on RNA, while the method introduced here can be used on other nanomachines and complexes, as well. The single fluorophore labeling schemes for RNA molecules and the statistical analysis to determine the true stoichiometry of the RNA subunits in case of incomplete labeling are discussed.

### Single fluorophore labeling of RNA

Single fluorophore labeling of each subunit is crucial step in direct counting of subunits using a photobleaching assay. Proteins are fusion tagged with a fluorescent protein, such as green fluorescent protein (GFP) and yellow fluorescent protein (YFP) (Chalfie *et al*. 1994; Zhang *et al*. 2002) to ensure single fluorophore labelling with high efficiency (Leake *et al*. 2006; Lee *et al*. 2009; McGuire *et al*. 2012; Simonson *et al*. 2010). Chemical synthesis can produce site specific labeled RNA strands with lengths up to 60–80 bases for single labeling of RNA molecules, single labeling can be achieved by *in vitro* transcription for longer RNA molecules using fluorescent adenosine monophosphates (AMPs) that can initiate the transcription, but cannot be incorporated for chain extension in a T7 RNA polymerase system with an Ø2.5 promoter (Huang *et al*. 2003; Li *et al*. 2005; Shu *et al*. 2007). Single labeling is achieved by annealing of the RNA with a short single-fluorophore labeled DNA/RNA oligo on an overhang on either side of the RNA molecule (Zhang *et al*. 2010a). A bipartite concept can also be used to produce singly labeled RNA molecules through assembly of two short RNA fragments. For shorter RNA, it is possible to make the single fluorophore labelling through chemical synthesis (Fang *et al*. 2005, 2008; Shu *et al*. 2011). In addition, single labeling can also be achieved by post-transcription chemical reaction of a RNA carrying functional group at the terminal ends with a fluorescent reagent (Hermanson 2013; Paredes *et al*. 2011) such as a thiol end-labeled RNA with commercially available fluorescent maleimide (Hermanson 2013), or through “click” chemistry of alkyne modified RNA with azide modified fluorophore (Bock *et al*. 2006; Paredes *et al*. 2011). The labeled RNAs are subject to further purification using gel filtration. The labeling efficiency of the RNA molecules can be obtained by measuring absorption at 260 nm and a maximum absorbance wavelength for the fluorophore and calculating the molar ratio between the fluorophore and the RNA molecule.

### Photobleaching assay and analysis of the photobleaching traces

Photobleaching of fluorophore causes a stepwise intensity to drop and direct counting of fluorophores is achieved by recording the total number of steps in the intensity change over time (Zhang *et al*. 2014).

### Counting of pRNA in phi29 DNA packaging motor

A multimeric pRNA ring gears the bacteriophage phi29 DNA packaging motor. The stoichiometry of the pRNA in the packaging motor has been debated for a long time (Guo *et al*. 1998; Ibarra *et al*. 2000; Morais *et al*. 2008; Shu *et al*. 2007; Simpson *et al*. 2000; Xiao *et al*. 2008; Zhang and Anderson 1998). Cryogenic electron microscopy (cryo-EM) studies from different laboratories have shown a hexameric (Ibarra *et al*. 2000) or a pentameric (Morais *et al*. 2008; Simpson *et al*. 2000) pRNA on motors. Biochemistry data have revealed that purified pRNA dimers and trimers are active in DNA packaging. This implies that the number of pRNA molecules on a motor would be a common multiple of two and three. Using single molecule photobleaching assay, the number of pRNA within one pRNA ring has been directly counted to be six (Shu *et al*. 2007).

In the photobleaching assay, each pRNA molecule was labeled with a fluorescent dye of Cy3 or Cy5 at its 5’ end via transcription with fluorescent AMP. The biological activities of the fluorescently labeled pRNA were tested before imaging for motor binding and DNA packaging to confirm that the pRNA retains its activity after labeling. The packaging motor was first assembled by mixing the labeled pRNA with phi29 DNA packaging motor in the presence of a buffer containing 100 mmol/L NaCl, 10 mmol/L MgCl_2_, and 50 mmol/L Tris at pH 8, and then isolated through 5%–20% sucrose gradient to remove the unbound pRNA (Shu *et al*. 2007). The complex was tested for biological activity then immobilized to the antibody-coated surface of the chamber for TIRF imaging with proper density. Transient fluorescent spots with an appearance of less than 10 frames were discarded in analysis. An oxygen scavenger system (0.5% β-D-glucose, 10 mmol/L β-mercaptoethanol, and GODCAT: 0.2% glucose oxidase and 0.25% catalase) was used to improve the photostability of dye during the imaging by slowing down the photobleaching caused by photo-oxidation (Rasnik *et al*. 2006). The specific antibody coating on the surface and the oxygen scavenger system helped to retain very low fluorescence background in the absence of fluorophores.

A laser beam of 532 nm was used for the excitation of Cy3 labeled pRNA. The dimension of the focused beam at the imaging interface was about 150 × 50 µm^2^. The samples were excited continuously and the laser power was adjusted to 5–8 mw to ensure slow photobleaching so that multiple fluorophores were not bleached simultaneously, making it difficult to resolve the steps. Sequential images were taken at a rate of 2.3–4.3 Hz with an exposure time of 0.2–0.4 s. Each fluorescent spot in the image (Fig. 2B) representing individual motor/pRNA complexes was analyzed by Andor iQ software (Andor Technology). Average intensity of a circled area around the fluorescent spot (yellow circle) was calculated with nearby background fluorescence subtracted (white circle) at each time point (Fig. 2B) and plots of average fluorescence intensity versus time were produced for individual fluorescent spots (Fig. 2C). Non-specific protein bindings were further reduced by surface blocking with bovine serum albumin (BSA) or coating with polyethylene glycol (PEG).

### Analysis of the photobleaching traces

The number of fluorophores within each spot was quantified by directly counting the number of photobleaching steps. As the environment of each fluorophore differs from each other within the sample chamber, the photobleaching step size may vary. A histogram with the distribution of step sizes was used to identify actual one-step-drops that come from multiple fluorophores. Furthermore, for fluorescent proteins, due to their intrinsic properties, their intensity fluctuates significantly during photobleaching, making it difficult to determine the step numbers. Mathematic filters, such as Chung-Kennedy filter (Chung and Kennedy 1991), were applied to the raw photobleaching traces to extract signals from noise, thus revealing distinctive steps from the traces (Coffman *et al*. 2011; Leake *et al*. 2006). A fully automated counting program has been further developed for automatic step detection in the photobleaching traces (McGuire *et al*. 2012). The greater number of fluorescent molecules in a complex, the more challenging the direct counting of steps will be. This is due to the increased possibility of synchronous photobleaching of multiple fluorophores, leading to the underestimation of the total steps. The distinction of the overlapping steps will also be challenging when more steps appear and fluctuation in step size occurs. With the current experimental setup, researchers were able to directly count up to 11 Cy3 photobleaching steps within one diffraction limited fluorescent spot empirically (Shu *et al*. 2007). A maximum of 15 steps was however predicted to be countable without mathematical extrapolation (Das *et al*. 2007). Alternatively, the number of fluorophores can be estimated by dividing the intensity of the fluorescent spot prior to photobleaching with that of a single fluorophore derived from the histogram of step size (Coffman *et al*. 2011; Leake *et al*. 2006; Taniguchi *et al*. 2010), although errors can occur due to the fluctuation of step size.

### Counting of pRNA in active packaging intermediates

The packaging motor was constructed using Cy3-labeled pRNA and Cy5-labeled genomic DNA to count the pRNA in active packaging intermediates (Shu *et al*. 2007). A non-hydrolysable ATP analog, γ-S-ATP, was used to stall DNA-packaging and isolated through a sucrose gradient. The packaging intermediate was confirmed by the co-detection of Cy3 and Cy5 signals within the same fluorescent spots. A control sample without packaging activity was also tested to confirm that the overlapped signals did not come from the accidental co-localization of the two fluorophores. Almost no co-localized Cy3 and Cy5 signals were observed for nonpackaging sample, suggesting that the Cy3-pRNA and Cy5-DNA would not co-exist within the same motor if DNA were not packaged.

### Simultaneous dual color photobleaching of differently labeled pRNA on phi29 DNA packaging motor

Wild type pRNA contains two essential interlocking loops with complementary sequences for dimerization. One pRNA ring can be differently labeled by re-engineering its loop sequences. For example, with the same letter in upper and lower cases representing the complementary sequences, a pRNA-Aa’ containing two complementary interlocking loops is self-efficient enough to form a dimer in solution, and subsequently a closed ring on the motor. However, a pRNA-Ab’ with unmatched loop sequences can only form a dimer, and subsequently a closed ring, with a partner pRNA-Ba’. Thus, the fluorescent pRNA ring was designed to have a Cy3 labeled pRNA-Ab’ paired with a Cy5 labeled pRNA-Ba’ (Shu *et al*. 2007). With the laser combiner and Dual-View^TM^ imager with an appropriate filter, the imaging system was able to detect fluorescence signals from the two differently labeled pRNA simultaneously. Sequential images were taken, and each image was processed through field-split function to overlay the signals in the Cy3 and Cy5 channels. Only the fluorescence spots that contained both Cy3 and Cy5 signals were analyzed for photobleaching to show the number of each fluorescently labeled pRNA inside an individual motor.

A sample of motor containing Cy3-pRNA only was examined and revealed that the crosstalk of Cy3 signal to Cy5 channel was approximately 15%, and cares were taken during the data analysis to ensure that the intensity drop from the bleed through was not counted for Cy5 quantification.

### Elucidating mechanism of pRNA ring assembly on the motor

**Figure 12:**
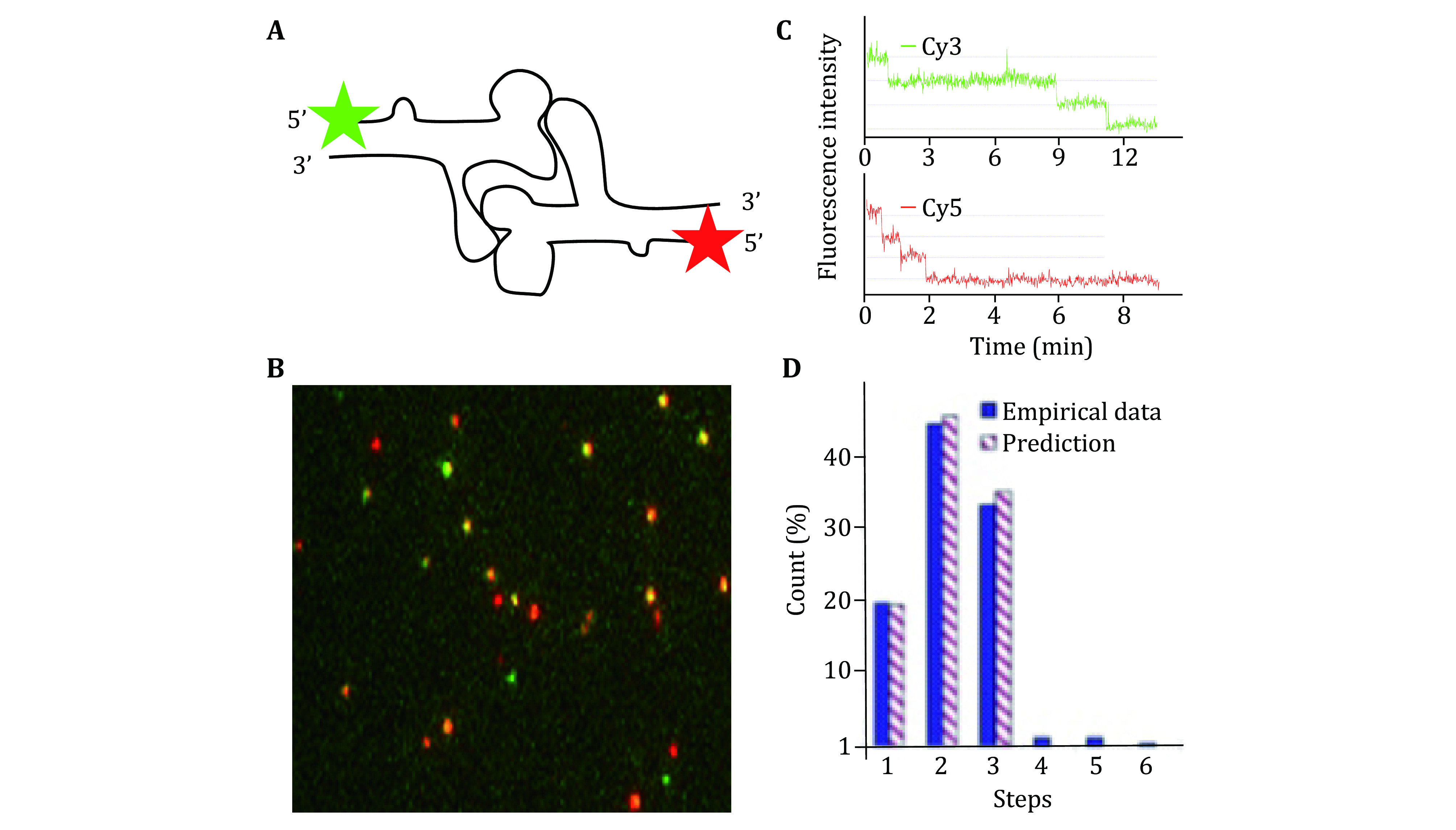
Dual color photobleaching of differently labeled pRNAs within the same motor/pRNA complex. **A** Design of dual-labeled pRNA for motor binding. **B** Typical overlaid fluorescence image of the dual color labeled motor/pRNA complexes. Green: Cy3; Red: Cy5; Yellow: Cy3/Cy5 overlay. **C** A dual-color photobleaching trace of motor/pRNA complex showing three Cy3-pRNA molecules and three Cy5-pRNA molecules on the same motor. **D** Comparison of the experimental histogram of photobleaching steps with the theoretical histograms for Cy3-pRNA based on 70% labeling efficiency. Adapted from Shu *et al*. (2007) with permission from European Molecular Biology Organization

Single molecule photobleaching study has revealed that the specificity and affinity in the motor/pRNA interaction are dependent on a static pRNA ring formation (Xiao *et al*. 2008) rather than conformational capture (Haller *et al*. 2011; Leulliot and Varani 2001) or induced fit (Cruz and Westhof 2009; Leulliot *et al*. 2001; Williamson 2000). Cy3-labeled pRNA-Aa’ has a much stronger affinity to the motor than pRNA-Ab’ that can only exist as monomers in solution, as demonstrated by the number of fluorescent spots observed in Fig. 12A. Analysis of their photobleaching traces also showed different distributions in copy numbers, with pRNA-Aa' having significantly more copies on the motor; while the majority of pRNA-Ab' showed only one copy, similar to that of free pRNA in solution (Fig. 12). Smaller mutant pRNA with the ability to form a closed ring was also found to be incapable of binding to the motor, showing much less binding compared to pRNA-Aa’ demonstrating size effect on the motor/pRNA interaction.

### Statistical analysis of photobleaching histograms

**Figure 13:**
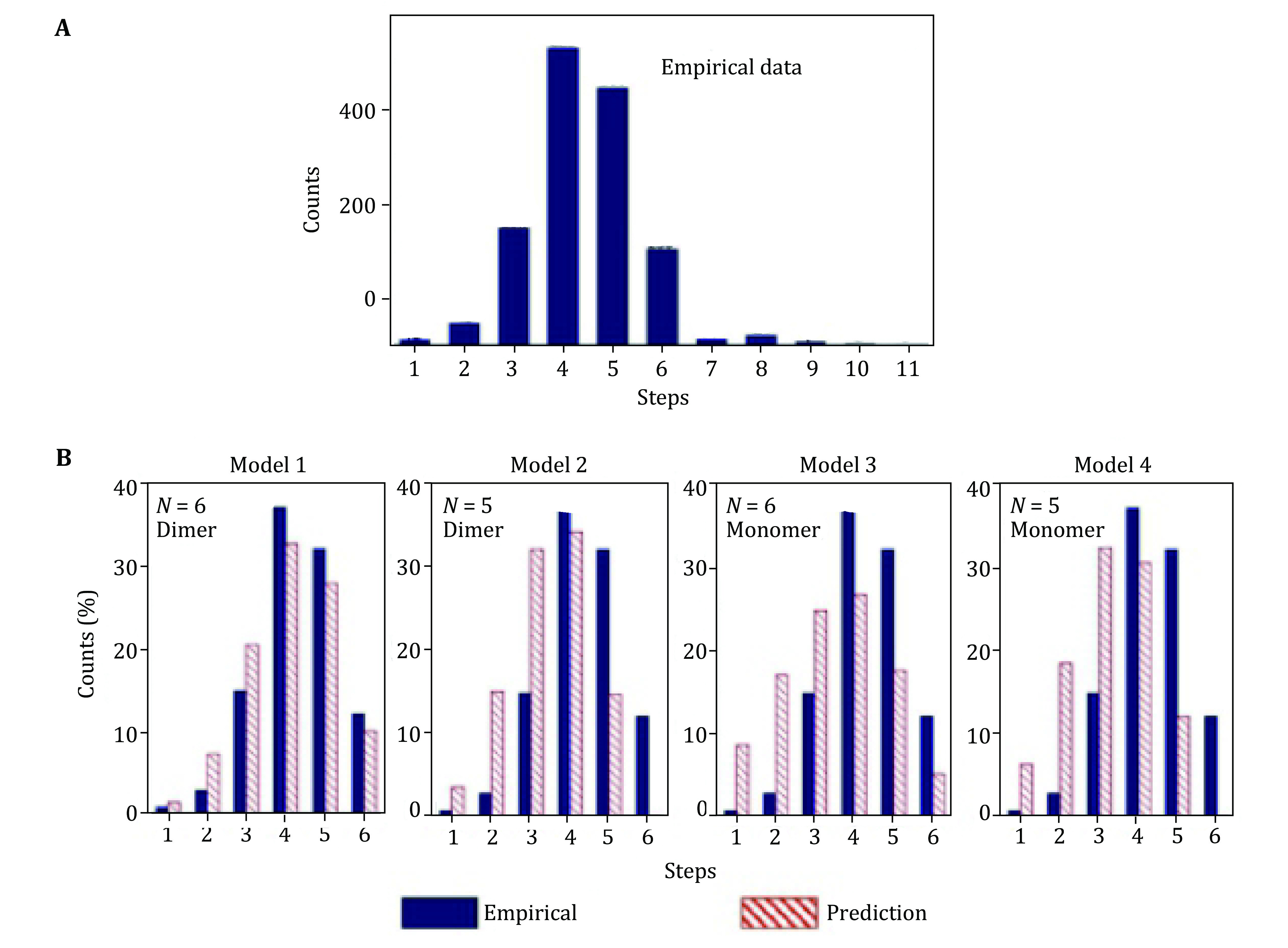
Statistical analysis to obtain the actual copy numbers of pRNA on motor. **A** Experimental histogram of photobleaching steps of motor/Cy3-pRNA complexes. **B** Fitting of the experimental data with different statistic models (Models 1–4) that were constructed based on 70% labeling efficiency for Cy3-pRNA. The best fit is the Model 1, indicating the presence of six pRNA molecules on the motor that were assembled from three dimers. Adapted from Shu *et al*. (2007) with permission from European Molecular Biology Organization

Fluorescent labeling of RNA could be incomplete so, the number of photobleaching steps in each experiment data do not directly reflect how many pRNA molecules are in that biological complex. The histogram of photobleaching steps of Cy3-pRNA in the motor/pRNA complex shows a distribution from 1 to up to 11 steps (Fig. 13). Statistical analysis involved labeling efficiency consideration.

The stoichiometry of pRNA is five or six on the motor was revealed by setting up models. Four statistical models were constructed based on different hypothesis as shown in Fig. 13B, in shaded columns. The models also considered different assembly pathways of pRNA onto a motor. The models were based on the following hypothesis (Shu *et al*. 2007):

Model 1: pRNA assembles into a hexamer on the motor from a pRNA dimer.

Model 2: pRNA initially assembles as a hexamer on the motor dimer. However, one pRNA shifts away and leaves a pRNA pentamer on the motor.

Model 3: pRNA assembles as a hexamer on the motor from a pRNA monomer.

Model 4: pRNA assembles as a pentamer on the motor from a pRNA monomer.

Mathematical formulas were developed for each model to predict the fractions of motor containing *i* copies of Cy3-pRNA, where 1 ≤ *i* ≤ 6, as only the Cy3-pRNA can be counted in the experiment. Mathematical details of the models and fittings of each model have been reported in detail previously (Zhang *et al*. 2014).

After evaluation of all four models, the best fit model was accepted; Model 1 gave the best fit with the empirical data, based on the fitting parameters (Shu *et al*. 2007). These results suggested that the pRNA ring on the motor is a hexamer assembled from pRNA dimers.

### Single molecule photo bleaching technology in single molecule counting of proteins

Stepwise photobleaching using fluorescence microscopy has been used for counting protein molecules. Like pRNA, protein counting is also based on the irreversible and stochastic loss of fluorescence from fluorescent proteins through a continuous light source.

**Figure 14:**
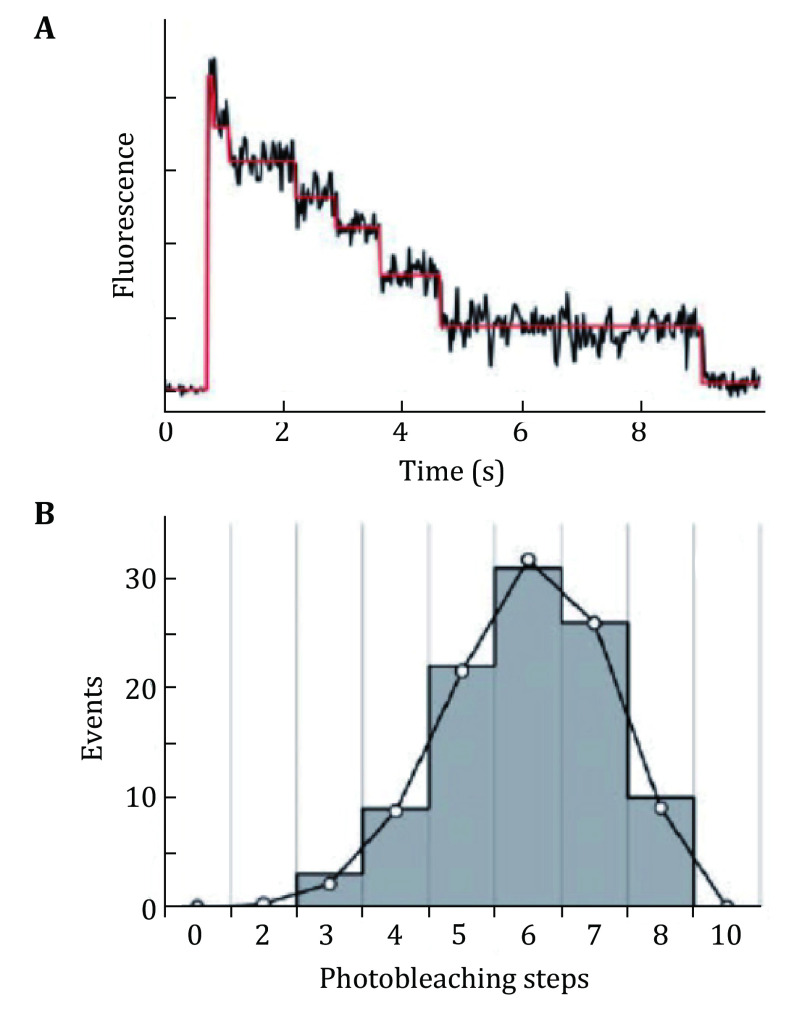
Single molecule photobleaching for stoichiometry of protein. **A** Stepwise single-molecule photobleaching of $ \propto {\rm{HL}}$ labeled with Alexa-647. **B** Histograms of all photobleaching steps. Adapted from Das *et al*. (2007) with permission from John Wiley & Sons

Leake *et al*. used SMPB to determine the stoichiometry of bacterial flagellar motor protein component Mot B (Leake *et al*. 2006). The authors calculated 22 copies of MotB subunits by the stepwise photobleaching of single GFP molecules. Das *et al*. prepared $ \propto {\rm{HL}}$ and leukocidin with single-cysteine mutants and one type of subunit within the pore complex was labeled with the fluorescent dye, Alexa-647 (Das *et al*. 2007). They collected the photobleaching steps in these complexes and determined the number of labeled subunits within $ \propto {\rm{HL}}$ and leucocidin from the distribution of bleaching steps (Fig. 14). The authors estimated the $ \propto {\rm{HL}}$ as a heptameric and leucocidin as an octameric protein complex by SMPB. Even the bleaching events or the step sizes can be counted directly, the calculation of determining stoichiometry becomes more complex when the system gets sophisticated. McGuire *et al*. automated the single subunit counting of membrane proteins in mammalian cells (McGuire *et al*. 2012). Using the automated method, the tetrameric and pentameric stoichiometry of GluK2 kainate and $ \propto $1-glycine neurotransmitter receptors respectively were determined. Yokota *et al*. used SMPB to determine the stoichiometry of nonhexameric helicase in *E. Coli* (Yokota *et al*. 2013). Mehta *et al*. measured dynamics and stoichiometry of a regulated enhancer-binding protein in live *E. coli* cells (Mehta *et al*. 2013). Fricke *et al*. utilized the single molecule localization microscopy (SMLM) to estimate the stoichiometry of membrane proteins by counting the number of photoblinkings in a fluorescently labeled protein (Fricke *et al*. 2015).

### Single molecule fluorescence to study folding of RNA structures

**Figure 15:**
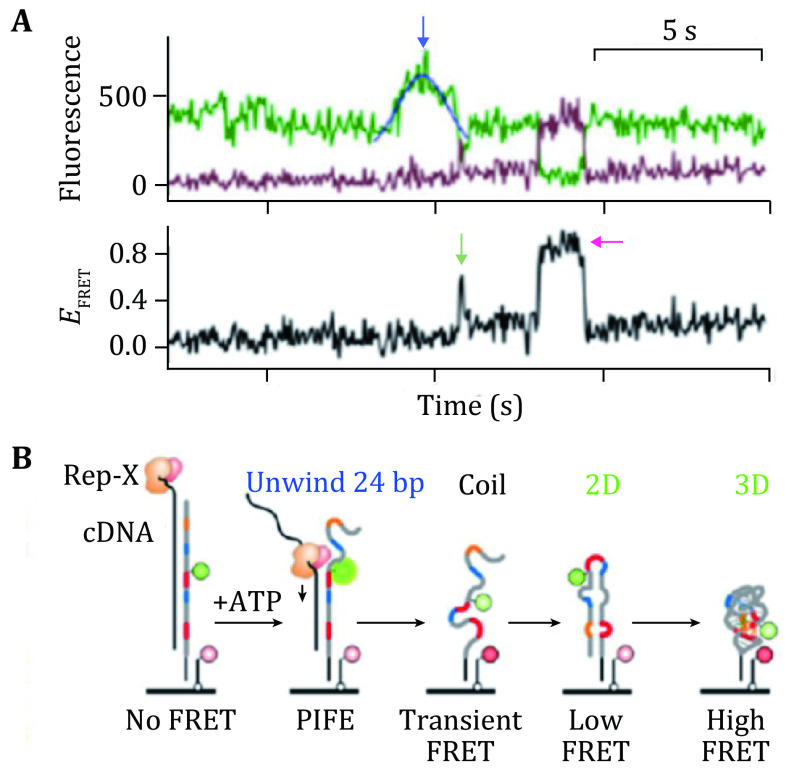
Real time observation of unfolding the duplex DNA−RNA hybrid and folding of RNA. **A** FRET trajectories showing the PIFE peaks (blue arrow) and the maiden 2D (green arrow) and 3D (magenta arrow) folding events. **B** Schematics of the “order-of-events” in **A**. Adapted from Hua *et al*. (2018) with permission from American Chemical Society

Naturally, RNA molecules fold into certain structures to perform their functions of gene expression or catalysis (Chen *et al*. 2018; Fallmann *et al*. 2017). The folding of RNA can be affected by many factors of ions and proteins through different mechanisms (Draper *et al*. 2005; Duss *et al*. 2019; Nguyen *et al*. 2019). Single molecule fluorescence microscopy has become an important tool to understand the process of RNA folding. Hua *et al*. studied co-transcriptional folding of RNA using pre-synthesized RNA (Hua *et al*. 2018) and found maiden secondary and tertiary folding pathways (Fig. 15A). They used single molecule FRET by strategically designed fluorescent labels (Fig. 15B).

## COMBINATION OF OTHER METHODS WITH OPTICAL TWEEZERS/smFRET

The impact of optical tweezers in biology has further expanded due to their integration with other experimental techniques. This includes their combination with multichannel microfluidics, which not only enhances experimental throughput but also provides *in-situ* control of more complex multistep biological processes (Enger *et al*. 2004; Gross *et al*. 2010). Most notably, the combination of optical tweezers with fluorescence techniques has enabled optical tweezers analyses to venture far beyond strictly mechanical measurements (Agate *et al*. 2004; Gross *et al*. 2010; Kong *et al*. 2011; Liu *et al*. 1995; Murade *et al*. 2009; Sirinakis *et al*. 2012). Recently optical tweezers have also been used in conjugation with nanopore technology (Keyser *et al*. 2006, 2009). This brings two powerful single molecule techniques together.

To simultaneously manipulate any image samples that exhibit fluorescence, optical tweezers can be built alongside a fluorescence microscope. Such instruments are particularly useful when it comes to studying single or small numbers of biological molecules that have been fluorescently labeled, or in applications in which fluorescence is used to track and visualize objects that are to be trapped. Combining fluorescence techniques with optical tweezers could increase the resolution of measurements in both temporal and spatial regimes. Combining these two techniques is challenging because of the faster photobleaching of fluorophore close to the trapping laser. Some researchers addressed this problem by separating the trapping light and the fluorescence excitation light either temporally or spatially. Dijk *et al*. developed a technique to combine fluorescence detection using a combination of optical tweezers and fluorescence microscopy and found enhanced photobleaching of the fluorophores (van Dijk *et al*. 2004). They proposed the enhanced photobleaching is caused by the absorption of a visible photon to form an excited-state which follows the absorption of a near-infrared photon used for trapping the light. Brau *et al*. tried to overcome the photobleaching problem by alternately modulating the optical trap and excitation beams so that simultaneous exposure of the fluorescent dye by trapping light and excitation light is prevented (Ma *et al*. 2019). They demonstrated a significant reduction in the rate of photobleaching effects on the single molecule fluorescence dye Cy3, which is considered highly susceptible to bleach quickly.

Hohng *et al*. studied the folding dynamics of DNA holiday junction (HJ) at lower force region by combining optical trap and single molecule fluorescence resonance energy transfer (smFRET) (Hohng *et al*. 2007). FRET describes the energy transfer between two chromophores, a donor and an acceptor. The FRET efficiency represents the probability of the energy transfer between the two chromophores. The emitted light from the donor has a possibility to excite the acceptor. In experiments, the donor will be excited with a light source, and the FRET efficiency can be determined by the decrease or increase in the fluorescence intensity for the donor and acceptor, correspondingly. To achieve this energy transfer, the emission spectrum of the donor dye should overlap well within the excitation spectrum of the acceptor dye. Since the efficiency of this energy transfer is inversely proportional to the sixth power of the distance between donor and acceptor, FRET measurements are extremely sensitive to small changes in distance. Based on this principle, scientists can determine the distance between two chromophores from the energy transfer efficiency measurements. In their experiments, they separated the trapping and fluorescence excitation beams in a confocal microscope spatially, nearly 13 µm. Confocal microscopy can increase the optical resolution and contrast of the images by using a spatial pinhole to block out-of-focus light during measurements; thereby ensuring that only the fluorescence light produced near the focal plane can be detected while the unfocused background light could be eliminated by the pinhole to reduce the background signals successfully. The authors of this work probed the HJ dynamics in response to pulling forces in three different directions and mapped the location of the transition states in the two-dimensional reaction landscape to deduce the global structure of the transient species populated during the HJ conformational changes.

A research group led by Nils G. Walter has extensively studied various nanoparticles including nano-devices and nano-engines (Dhakal *et al*. 2016; Valero *et al*. 2018). Dhakal *et al*. designed a DNA actuated enzyme nano reactor which was evaluated by smFRET, atomic force microscopy (AFM) and molecular dynamic simulations (Dhakal *et al*. 2016). In this study, they advanced the tweezer closure and tweezer-actuated enzyme function by modifying the actuator hairpin and the architectural holiday junction sequence. In another study by Valero *et al*., researchers developed a biohybrid nano-engine powered by nucleotide triphosphates (NTPs) that moves unidirectionally over a DNA nanotube. This result was characterized by smFRET and AFM (Valero *et al*. 2018). The engine made of an engineered T7 RNA polymerase attached to a dsDNA nanoring by a zinc finger domain was catenated to a rigid rotating dsDNA wheel. This motor produced a repetitive RNA sequence that remain attached to the engine and was used to guide its movement along predefined tracks of ssDNA on a DNA nanotube.

**Figure 16:**
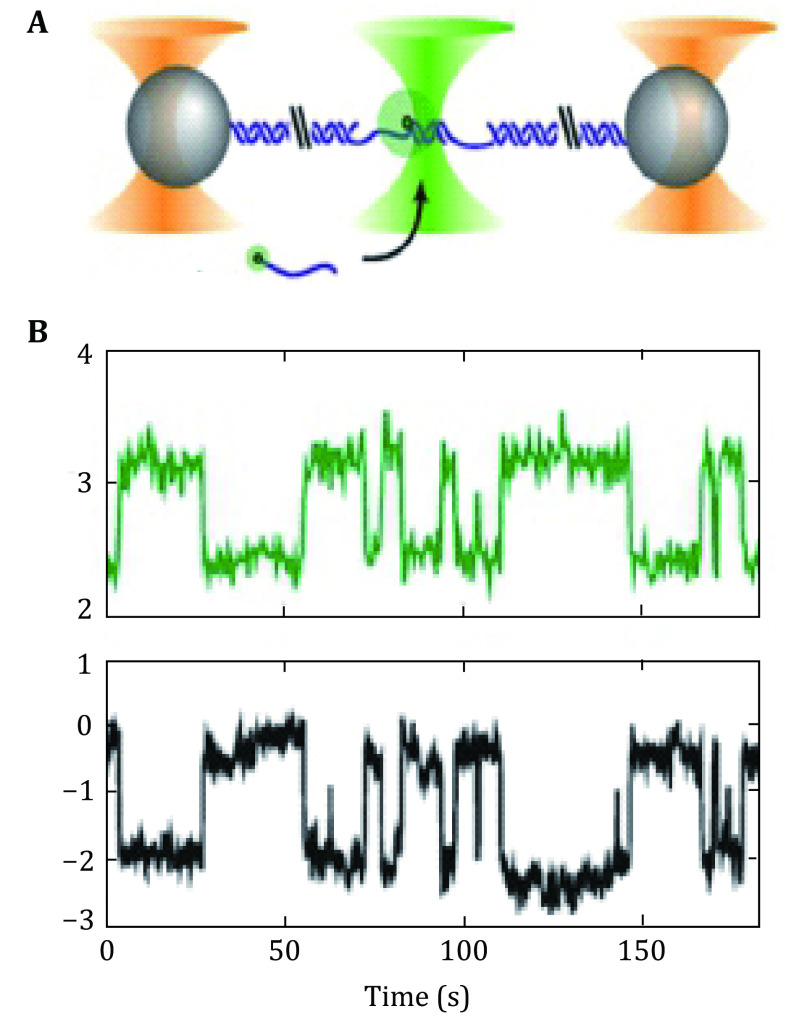
Combination of experiments with high-resolution optical trap and fluorescence (not to scale). **A** Left panels: polystyrene microspheres (grey) are held in optical traps (orange cones), tethered by an engineered DNA molecule (blue) containing a variable central segment flanked by long double-stranded DNA (dsDNA) handles. Fluorophores are excited by a green laser (green cone). **B** Time traces showing simultaneous measurement of fluorescence and tether extension by oligonucleotide hybridization with short oligonucleotides (blue line) labeled with a fluorophore (green disk) bind and unbind to a complementary ssDNA section in the center of the tethered DNA. The fluorescence and change in tether extension upon hybridization are recorded simultaneously. Adapted from Chuang *et al*. (2019) with permission from American Chemical Society

Comstock *et al*. combined confocal microscopy with optical tweezers (Chuang *et al*. 2019; Comstock *et al*. 2011). They minimized the photobleaching effect by switching on and off the excitation light and trapping light alternatively using acousto-optic modulators. They studied the 9-nt DNA annealing and melting processes and how it changes under external force with angstrom level resolution (Whitley *et al*. 2017). Chuang *et al*. simultaneously measured angstrom-scale changes in tether extension and fluorescence signals (Chuang *et al*. 2019) (Fig. 16A, 16B).

## FUTURE PROSPECTIVE

A typical feature of the living system is motion. The lack of synchronicity of motion machines in a living system makes it challenge to image the motion process with high resolution. Thus, single molecule technology is especially useful for the real-time study on the mechanism of motion machines, such as viral DNA packaging motor, or the ATPase type biomotors. Single molecule techniques have enabled the study of biomolecules (one molecule) at a time and made it possible to investigate the properties of individual complexes or molecular processes. The most common optical instrumentation used in single molecule studies are optical tweezers and smTIRF. Optical tweezers are the force-based technique that has revealed many molecular mechanisms as well as physical properties of biomolecules. The analysis of RNA using optical tweezer has led to the discovery of the rubbery or amoeba property of RNA nanoparticles for compelling vessel extravasation to enhance tumor targeting and for fast renal excretion (Ghimire *et al*. 2020) thus solving two previous puzzles: (1) Why RNA nanoparticles can target tumor passively and efficiently, since the rubber- and amoeba-like deformation property enables RNA nanoparticles to squeeze themselves out of the newly produced leaky blood vessel under the blood pressure? (2) Why RNA nanoparticles remain non-toxic since they can be rapidly cleared from the body via renal excretion into urine with little accumulation in the healthy organs in the body. The elucidation of the rubbery mechanism leads to the believing of RNA as nontoxic materials for cancer therapy. Single molecule photobleaching by smTIRF allows for the direct counting of biomolecules in a straightforward manner. This technique has been widely used for single molecule counting of RNA in phi29 DNA packaging motor (Shu *et al*. 2007). The technology has subsequently extended to the counting of the number of subunits or components in biological machines composed of protein, DNA, and other macromolecules. RNA, protein, or DNA molecules in nanoparticles can be counted, by combining it with single fluorophore labeling and statistical analysis. Single molecule photobleaching technology will provide insights into the RNA stoichiometry in nanoparticles, which is essential to a better understanding of various RNA functions, as well as better characterizations of RNA nanoparticles for nanomedicine. With better precision, sample preparation, and computation will allow these single molecule methods to reveal biomolecular mechanisms in detail as well as may help to develop ultra-sensitive sensors to detect biomarkers and chemical species to be used in diagnostic and forensic.

## Conflict of interest

Chiran Ghimire and Peixuan Guo declare that they have no conflict of interest.
